# *Cis* P-tau is induced in clinical and preclinical brain injury and contributes to post-injury sequelae

**DOI:** 10.1038/s41467-017-01068-4

**Published:** 2017-10-17

**Authors:** Onder Albayram, Asami Kondo, Rebekah Mannix, Colin Smith, Cheng-Yu Tsai, Chenyu Li, Megan K. Herbert, Jianhua Qiu, Michael Monuteaux, Jane Driver, Sandra Yan, William Gormley, Ava M. Puccio, David O. Okonkwo, Brandon Lucke-Wold, Julian Bailes, William Meehan, Mark Zeidel, Kun Ping Lu, Xiao Zhen Zhou

**Affiliations:** 1 Division of Translational Therapeutics, Department of Medicine, Beth Israel Deaconess Medical Center, Harvard Medical School, 330 Brookline Avenue, CLS 0408, Boston, MA 02215 USA; 2 Department of Medicine, Beth Israel Deaconess Medical Center, Harvard Medical School, 330 Brookline Avenue, Boston, MA 02215 USA; 3 Cancer Research Institute, Beth Israel Deaconess Medical Center, Harvard Medical School, 330 Brookline Avenue, CLS 0408, Boston, MA 02215 USA; 4 Division of Emergency Medicine, Children’s Hospital Boston, Harvard Medical School, 300 Longwood Ave, Boston, MA 02115 USA; 5 Department of Neuropathology, University of Edinburgh, 49 Little France Crescent, Edinburgh, EH16 4SB UK; 6 Geriatric Research Education and Clinical Center, VA Boston Healthcare System, Harvard Medical School, 150S Huntington Ave, Boston, MA 02130 USA; 7 Department of Neurosurgery, Brigham and Women’s Hospital, Harvard Medical School, 75 Francis Street, Boston, MA 02115 USA; 8 Department of Neurosurgery, University of Pittsburgh Medical Center, 200 Lothrop St, Pittsburgh, PA 15213 USA; 9 Department of Neurosurgery, West Virginia University, Suite 4300, Health Sciences Center, PO Box 9183, Morgantown, WV 26506 USA; 10 Department of Neurosurgery, NorthShore University Health System, University of Chicago, Pritzker School of Medicine, 3rd Floor Kellogg, Evanston, IL 60637 USA; 11 Micheli Center for Sports Injury Prevention, Division of Sports Medicine, Children’s Hospital Boston, Harvard Medical School, 319 Longwood Avenue, Boston, MA 02115 USA

## Abstract

Traumatic brain injury (TBI) is characterized by acute neurological dysfunction and associated with the development of chronic traumatic encephalopathy (CTE) and Alzheimer’s disease. We previously showed that *cis* phosphorylated tau (*cis* P-tau), but not the *trans* form, contributes to tau pathology and functional impairment in an animal model of severe TBI. Here we found that in human samples obtained post TBI due to a variety of causes, *cis* P-tau is induced in cortical axons and cerebrospinal fluid and positively correlates with axonal injury and clinical outcome. Using mouse models of severe or repetitive TBI, we showed that *cis* P-tau elimination with a specific neutralizing antibody administered immediately or at delayed time points after injury, attenuates the development of neuropathology and brain dysfunction during acute and chronic phases including CTE-like pathology and dysfunction after repetitive TBI. Thus, *cis* P-tau contributes to short-term and long-term sequelae after TBI, but is effectively neutralized by *cis* antibody treatment.

## Abstract (web summary)

Induction of the *cis* form of phosphorylated tau (*cis* P-tau) has previously been shown to occur in animal models of traumatic brain injury (TBI), and blocking this form of tau using antibody was beneficial in a rodent model of severe TBI. Here the authors show that *cis* P-tau induction is a feature of several different forms of TBI in humans, and that administration of *cis* P-tau targeting antibody to rodents reduces or delays pathological features of TBI.

## Introduction

Traumatic brain injury (TBI) is the leading cause of death and disability among people under the age of 45 years^1^. Worldwide, 10 million deaths and/or hospitalizations annually are directly attributable to TBI and an estimated 57 million people are currently living with the consequences of TBI^2^. In the United States, 1.6−3.6 million athletes sustain TBI each year^3^, ~20% of 2.3 million troops deployed to Iraq and Afghanistan experienced a TBI^4, 5^ and visits for TBI to Emergency Departments in the US have increased eightfold more than the total increase between 2006 and 2010^6^. Diverse mechanisms of TBI, including repetitive mild TBI (rmTBI), as seen in collision sports^3^, and single moderate/severe TBI (ssTBI), as seen in military blasts^4, 5^ or motor vehicle accidents, cause acute and potentially long-lasting neurological dysfunction. TBI is also a major risk factor for neurodegenerative diseases, such as chronic traumatic encephalopathy (CTE)^7–10^, Alzheimer’s disease (AD)^11–14^, and Parkinson’s disease^15^. However, these neurodegenerative disorders occur many years or decades after TBI, and the mechanisms leading from acute TBI to chronic neurodegeneration are virtually unknown^7–10, 16^. Moreover, the search for targeted pharmacologic interventions has been nearly universally unsuccessful in mitigating the short-term or long-term outcomes of TBI^17, 18^. Establishing the causal link between TBI and neurodegenerative diseases could lead to critically needed targeted therapies.

Tau pathology is a common feature of several neurodegenerative disorders, together known as tauopathies^19, 20^. Neurofibrillary tangles composed of phosphorylated tau are the neuropathological signature of CTE found at autopsy in the brains of boxers, American football players, and blast-exposed veterans^7–10, 21, 22^. Tau tangles are also a hallmark of AD^19, 20^, and the tau isoform and phosphorylation profiles of tangles purified from CTE brains and AD brains are indistinguishable^23^. Tau in tauopathies is often hyperphosphorylated on Ser or Thr residues preceding a Pro residue resulting in disruption in its microtubule function and alterations in its protein stability, eventually leading to tau aggregation and tangle formation^19, 20^. In addition, other posttranslational modifications such as truncation, sumoylation and acetylation have been shown to affect tau function and contribute to the development of tau pathology^19, 20^. Various aspects of tau pathology, including tau hyperphosphorylation, oligomerization, aggregation, and tangle-like formation have been observed in animal models of tauopathies, without the development of mature tau tangles. Furthermore, tau pathology spreads through the brain^24, 25^. Moreover, although immunization with full-length tau protein has been shown to induce histopathologic features of Alzheimer disease and tauopathies^26^, active or passive immunization targeting certain tau fragments or pathological tau epitopes has shown some benefit against tauopathy without adverse effects, with some in early clinical trials^27, 28^. Thus, tau may offer a promising therapeutic target for tauopathies.

While tau tangle pathology has long been described in CTE and AD, such pathology following a single TBI is less well-described. Earlier case reports have described AD-like tangle pathology after a single, severe TBI followed by onset of dementia^29, 30^ and a more recent study has found tau tangles in ~30% of 39 human survivors 1 year or more from a single moderate to severe TBI^31^. However, there is not obvious tau pathology in 45 patients who died acutely (up to 1 month) following a single TBI^32^. The presence of tau pathology after TBI in preclinical models has been inconclusive. For example, tau phosphorylation and oligomers are detected acutely after open head, severe TBI in some rat models^33, 34^. Furthermore, tau phosphorylation and tangle-like pathologies have been observed many months after closed head repetitive TBI in some reports^34–36^, but not in others^37, 38^. Thus, the role of tau pathology in linking TBI to neurodegeneration is unclear.

We have previously identified a proline isomerase, Pin1 that inhibits the development of tau pathology and neurodegeneration in AD by converting the phosphorylated Thr231-Pro motif in tau **(**P-tau**)** from the pathogenic *cis* conformation to the physiologic *trans* conformation^39–47^. We developed polyclonal and monoclonal antibodies able to specifically distinguish and eliminate these two protein conformations and identified *cis* P-tau as a precursor of tau pathology and an early driver of neurodegeneration^48–50^. Within hours after closed head injury in mouse models, or following neuron stress in vitro, neurons produce *cis* P-tau prior to tau oligomerization and aggregation, which causes and spreads axonal pathology by a pathogenic process which we term cistauosis, including disruption of axonal microtubules and transport system, eventually leading to neuronal death^48^. Cistauosis is effectively blocked in vitro and in vivo by *cis* P-tau monoclonal antibody (*cis* mAb)^48^. Specifically, *cis* mAb prevents extracellular *cis* P-tau from spreading, and also enters neurons via Fcγ receptors to target intracellular *cis* P-tau for TRIM21-mediated proteasome degradation, rendering *cis* P-tau resistant to degradation and dephosphorylation to be degradable^48–50^. The importance of TRIM21 in degrading tau immunocomplexes has been confirmed^51^. Treating severe TBI mice with *cis* mAb not only eliminates early *cis* P-tau accumulation after injury and blocks cistauosis, but also prevents the later development of tau tangles and brain atrophy^48^. These results reveal that *cis* P-tau is critical for the development of axon pathologies, offering a potential link between TBI and neurodegeneration, and suggest *cis* P-tau antibody might be used to block tau pathology and prevent neurodegeneration after TBI^48–50^. The therapeutic potential of *cis* P-tau antibody is further supported by the findings that tau knockout prevents axon pathology and memory deficits after repetitive mild TBI in mice^52^ and that immunotherapy can effectively remove toxic proteins in the brain, even in patients with mild cognitive impairment^27, 28, 53, 54^.

It is still unknown whether *cis* P-tau is induced acutely after TBI in humans, especially given a prior study that showed no tau pathology was not found in the brains of 45 patients who died within 2 months after TBI^32^. Moreover, since there are many other short-term and long-term pathological and functional outcomes of TBI^7–10^, it is not known whether treatment with *cis* P-tau antibody would mitigate these outcomes. These questions are important for elucidating the molecular mechanisms underlying TBI and its consequences, and for understanding the potential impact of *cis* P-tau targeted therapy on TBI.

To demonstrate the importance of *cis* P-tau to acute and chronic TBI in humans, we examined *cis* P-tau in brains and cerebrospinal fluid acutely after severe TBI in humans and at chronic time points after injury in CTE brains from athletes with exposure to rmTBI. We found that severe TBI in humans due to diverse mechanisms (including motor vehicle accidents, assaults or falls) acutely and robustly induces toxic *cis* P-tau in cortical axons and cerebrospinal fluid, correlating with traumatic axonal injury and functional outcome 1 year after injury. In CTE brains with more remote TBI exposure, *cis* P-tau is widespread in the brain and correlates with various neurodegenerative pathologies. These results suggest that *cis* P-tau might also be involved in the development of other short-term and long-term outcomes of severe and repetitive TBI. To test this hypothesis, we utilized established mouse models of severe and repetitive mild TBI and elimination of *cis* P-tau induction and spreading using a neutralizing *cis* mAb to examine its impact on pathological and functional outcomes after injury. Indeed, elimination of *cis* P-tau effectively blocks the development and progression of not only tau pathology, but also an array of TBI-related neuropathological and functional outcomes during acute and chronic phases. Moreover, we have provided direct evidence that rmTBI in mice is sufficient to induce a range of widespread neuropathological features and functional deficits resembling those found in human CTE. More importantly, these CTE-like neuropathology and dysfunction after rmTBI are potently mitigated by eliminating *cis* P-tau using *cis* mAb. Thus, *cis* P-tau is a causative agent for the development and progression of a range of short-term and long-term outcomes of ssTBI or rmTBI, but can be effectively blocked in rodent models by *cis* mAb treatments. These results suggest that *cis* mAb may be further developed for early diagnosis and treatment of TBI and prevention of CTE and AD later in life in humans.

## Results

### Axonal injury and *cis* P-tau induction in clinical severe TBI

**Fig. 1:**
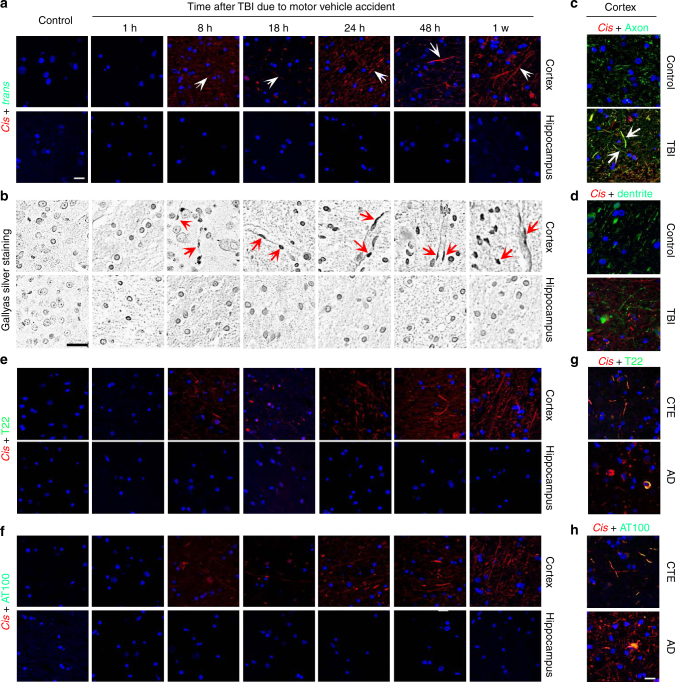
Severe TBI in humans due to motor vehicle accidents, assaults or falls induces prominent axonal injury and axonal *cis* P-tau induction in the cortex. **a**, **b** Severe TBI in humans induces early *cis* P-tau induction and axonal injury in the cortex. Whereas neither *cis* nor *trans* P-tau nor axonal injury was detected in normal brains or 1 h after TBI due to motor vehicle accident, robust *cis* P-tau and axonal injury, but not *trans* P-tau were detected in the cortex, but not in the hippocampus, as early as 8 h after motor vehicle accident, as detected by double IF, followed by isotype-specific secondary antibodies **a** or Gallyas silver staining **b**. Microscope images correspond to the cortex and hippocampus of control and TBI patients. TBI cases due to falls and assaults are shown in Supplementary Fig. 1. The number of TBI patients is 14. White arrows point to *cis* P-tau localization to axons; Red arrows point to axonal bulb. White scale bars, 20 µm and black scale bars, 40 µm. **c**, **d** *cis* P-tau (red) is diffusely co-localized (white arrows) with the axon marker tau (green) **c**, but not the dendrite marker MAP2 (green) **d** in human TBI cortex, with little *cis* in control, as detected by double IF, followed by confocal microscopy. **e**−**h** *Cis* P-tau is robustly induced after TBI in the absence of tau oligomers or tangles. Cortical sections of severe human TBI due to motor vehicle accidents were doubly immunostained with *cis* mAb (red) and T22 (tau oligomers) **e**, **g** or AT100 (tau tangles) **f**, **h**, followed by confocal microscopy. Normal controls as well as CTE and AD brains were used as negative and positive controls, respectively. Of note, *cis* mAb partially co-localized with T22 or AT100 in CTE or AD brains, but not in acute TBI brains

We first examined whether and where *cis* P-tau is induced acutely after TBI in human brains by performing immunostaining on cortical and hippocampal tissues of 14 patients 3−67 years of age who died from a TBI-related death. These patients had documented survival time for 1 h to 1 month after injury and primary injury mechanisms included motor vehicle accidents (6 cases), assaults (4 cases), falls (3 cases), or unknown cause (1 case) (Supplementary Table 1). Neither *cis* nor *trans* P-tau was detected on controls (people who died without CNS causes or diseases) or 1 h after TBI (Fig. 1a), as shown previously in normal human and mouse brains^48^. However, robust *cis*, but not *trans*, P-tau was readily and diffusely detected in the cortex, but not in the hippocampus, as early as 8 h after TBI in all 13 TBI patients examined, with variable intensity (Fig. 1a, Supplementary Fig. 1). *Cis* P-tau in the cortex was mainly localized to axons diffusely, but not in dendrites (Fig. 1c, d). Notably, traumatic axonal injury, one of the most common and important pathological features of closed head injury^55^, was also obvious in the cortex, but not in the hippocampus, as demonstrated by Gallyas silver-positive axonal bulbs (Fig. 1b), as previously described^56, 57^. However, as documented by well-established antibodies, none of these acute TBI samples had obvious tau oligomers (as detected by oligomeric tau antibody T22), early tau tangles (AT8 antibody), late tau tangles (AT100 antibody), amyloid beta peptide aggregation (Aβ antibody), or TDP-43 pathologies (TDP-43 antibody) in the cortex or hippocampus (Fig. 1e, f, Supplementary Table 1), in contrast to CTE and AD brains where *cis* P-tau partially co-localized with T22 and AT100 (Fig. 1g, h). However, acute TBI, especially at survival day 7, did induce a tendency toward increased staining intensity of ionized calcium-binding adapter molecule 1 (Iba1) positive microglia in the cortex, however the increase in intensity was not significant compared to controls, in contrast to CTE brains (Supplementary Fig. 2). Thus, severe TBI in humans acutely and prominently induces *cis* P-tau, which is most notable in the axons and is associated with axonal injury. There is no evidence of tau oligomers or tangles, gliosis, Aβ or TDP-43-related pathologies. In this series of TBI patients who survived up to 1 month after injury, both *cis* P-tau and axonal injury are limited to the cortex, but do not reach to the hippocampus. This pattern has been shown previously after severe TBI in mouse models^48^.

### CSF *cis* P-tau correlates well with outcome in TBI patients

**Fig. 2:**
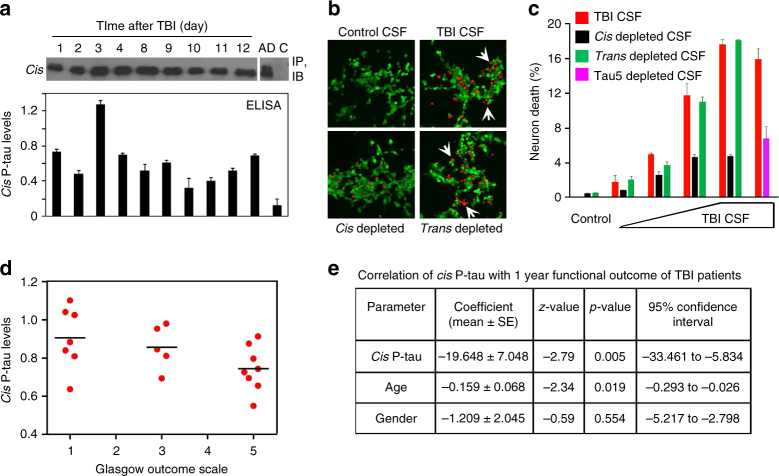
*Cis* P-tau in the CSF of severe TBI patients is neurotoxic and correlates with clinical outcome at 1 year after injury. **a** Detection of *cis* P-tau in the CSF of a TBI patient was performed by subjecting control CSF or CSF specimens collected from EVD at different times after TBI due to jumping into an oncoming train to immunoprecipitation with *cis* mAb, followed by immunoblotting with rabbit anti-tau mAb (top panel) or to direct ELISA with *cis* mAb in OD at 450 nm (bottom panel). Post-mortem Alzheimer’s patient CSF (AD) and control CSF were used as positive and negative controls, respectively. **b**, **c** Addition of TBI CSF to neurons induces dose-dependent cell death in recipient cells, which are fully rescued by immunodepletion with *cis*, but not *trans* mAb. Human control and TBI CSF specimens were added to culture media of SY5Y neurons for 3 days, followed by the live (green)/dead (red, white arrows) cell assay. For immunodepletion, *cis* or *trans* mAb was incubated with TBI CSF to deplete *cis* or *trans* p-tau before adding to cells. *n* = 3. **d**, **e** *Cis* P-tau levels of CSFs taken day 4 to 6 days after injury of acute TBI patients were measured by direct ELISA and an ordered logistic regression was used to model 1 year GOS as the outcome and *cis* P-tau level in OD at 450 nm as the main predictor, controlling for age, gender and initial Glasgow Coma Scale. Supplementary Table 2 shows demographic information for the subjects. *n* = 20

To confirm this early *cis* P-tau induction after TBI in humans, we obtained cerebrospinal fluid (CSF) samples collected from an external ventricular drain (EVD) placed in patients with severe TBI as part of their routine clinical care. CSF *cis* P-tau was assayed using immunoprecipitation with *cis* mAb, followed by immunoblotting with tau antibody E178, or using direct ELISA with *cis* mAb. Both assays readily detected *cis* P-tau in CSF samples obtained on different acute days after TBI, with generally similar results (Fig. 2a, Supplementary Fig. 13). *cis* P-tau in human TBI CSFs migrated as a single major band of 50 kDa on SDS-gels, similar to those observed in TBI mouse brain samples^48^ (Figs. 4c, 5b, and 6b), although additional slower migrated bands were also observed in post-mortem AD CSFs (Fig. 2a, Supplementary Fig. 13). The presence of CSF *cis* P-tau in human patients was further confirmed by a functional assay in vitro. Since *cis* P-tau is able to enter neuroblastoma SY5Y cells and induce cell death after being added to culture media^48^, we added human TBI CSF or control CSF samples to culture media of growing SY5Y cells for 3 days; we then assayed cell death using the live/dead cell assay, as described^48^. TBI CSF samples but not control CSF samples induced neuron death in a dose-dependent manner (Fig. 2b, c). Neuron death was significantly blocked by immunodepleting *cis*, but not *trans* P-tau using the respective mAbs prior to being added to culture media (Fig. 2b, c), supporting the specificity of *cis* P-tau-induced neuron death^48^. Depletion of total tau using Tau5 mAb also prevented the ability of human TBI CSFs from inducing neuron death (Fig. 2c), as shown previously for TBI brain lysates to induce neuron death^48^.

Next, to examine the significance of *cis* P-tau in human TBI, we used direct ELISA to measure CSF *cis* P-tau levels between days 4 and 6 after injury in 26 patients with severe TBI (GCS <8) from two tertiary care centers who had undergone EVD placement as part of their routine clinical care. We examined the correlation of acute *cis* P-tau expression with the Glasgow Outcome Scale (GOS) score in 20 patients with 1 year of follow-up (Fig. 2d, e, Supplementary Table 2). We used an ordered logistic regression model with 1 year GOS score as the outcome and CSF *cis* P-tau level as the main predictor, controlling for age, gender, and initial Glasgow Coma Scale (initial injury severity) score. In the multivariable model, there was a significant, inverse relationship between CSF *cis* P-tau levels and the GOS outcome (*p* = 0.005) (Fig. 2d, e). Although further studies are needed to establish utility of CSF *cis* P-tau as a biomarker in TBI, these results show that severe TBI in humans acutely induces *cis* P-tau in the cortex and CSF, correlating with traumatic axonal injury and clinical outcome.

### *Cis* P-tau found in deeper brain regions in CTE patients

**Fig. 3:**
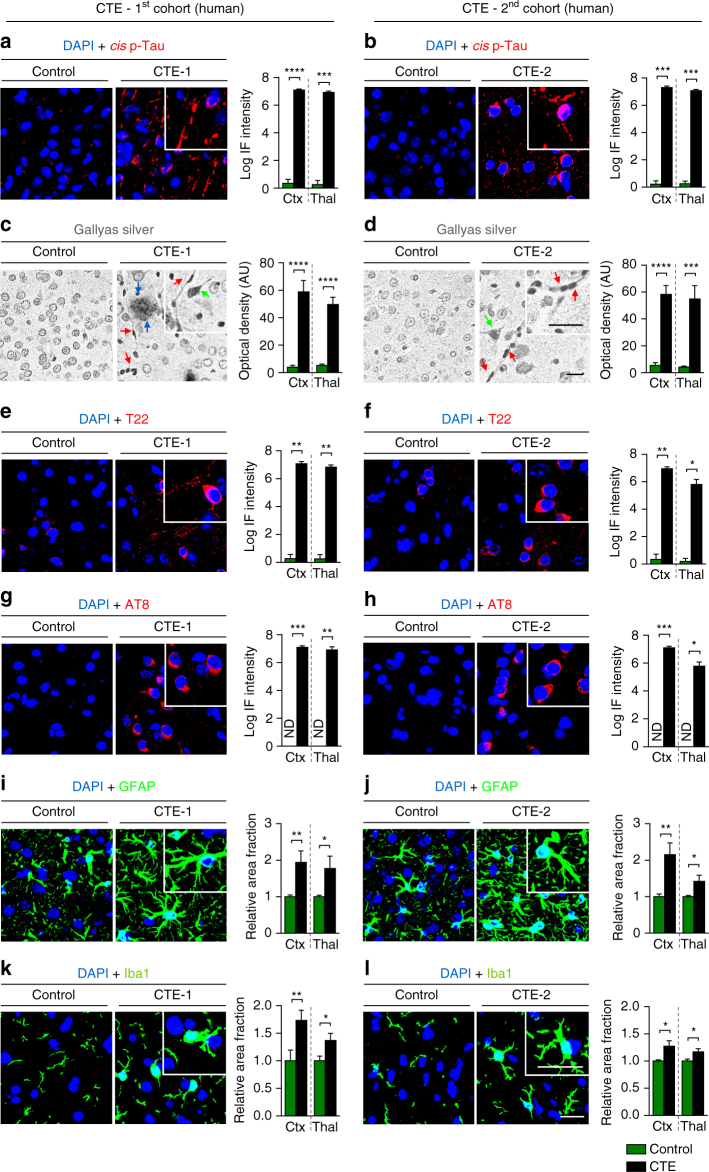
*Cis* P-tau is expressed in both the cortex and deep brain regions and correlates with various neuropathological features in CTE patients. CTE brain sections of the cortex and thalamus from eight collision sport athletes under ages of 75 from two independent sources and age-matched controls (Supplementary Table 3) were subjected to immunostaining or Gallyas silver staining, followed by confocal microscopy and light microscopy, respectively, to detect *cis* P-tau and various CTE pathologies. *Cis* P-tau staining **a**, **b** was correlated with the presence of axonal pathology, as detected by Gallyas silver staining **c**, **d**, tau oligomerization (T22) **e**, **f**, early tangles (AT8) **g**, **h**, astrogliosis (GFAP) **i**, **j** and microgliosis (Iba1) **k**, **l** in two different brain regions. Inset images are the high magnification image of selected area denoted by the white. Of note, there was not detectable signals when secondary antibodies were used alone (data not shown). Scale bar, 40 μm. Different types of pathologies in CTE brains revealed by Gallyas silver staining: senile plaque (blue arrow); neurofibrillary tangles (green arrow); axonal bulb (red arrow). Ctx, parasagittal cortex; Thal, thalamus; ND, not detectable; NS, not significant. Images were quantified and results are shown as means ± S.E.M. and *p*-values calculated using unpaired two-tailed parametric Student’s *t*-test. *p < 0.05, **p < 0.01, ***p < 0.001, ****p < 0.0001

Given the correlation between *cis* P-tau and 1-year clinical outcome of patients with TBI, we next asked if *cis* P-tau is associated with chronic TBI pathologies. To address this question, we examined the relationship between *cis* P-tau and other secondary neuropathologies. We obtained the post-mortem brains of eight athletes involved in collision sports who were <75 years of age and met criteria for CTE, from two independent sources, and compared them to age-matched controls (Supplementary Table 3). In CTE brains, we found that *cis* P-tau was detected not only in the cortex, but also in deeper regions, such as the thalamus (Fig. 3a, b), consistent with our previous findings in mouse models showing that *cis* P-tau spreads across different brain regions with time after impact-induced or blast-induced TBI^48^. Moreover, *cis* P-tau was correlated with the presence of a range of the neuropathological features of CTE including axonal pathology (Gallyas silver staining) (Fig. 3c, d; Supplementary Fig. 3a, b), tau oligomerization (T22) (Fig. 3e, f), early tangles (AT8) (Fig. 3g, h), and late tangles (AT100) (Supplementary Fig. 3c, d). Furthermore, we observed other secondary pathologies including glial fibrillary acidic protein (GFAP)-positive astrocytes (Fig. 3i, j) and Iba1-positive microglia (Fig. 3k, l) (two common indicators of chronic neuroinflammation^58^), TDP-43 pathology (especially with increased mislocalization spreading from the nucleus to cytoplasm (Supplementary Fig. 3e−g) and demyelination as detected by the oligodendroglial cell (myelin producing) marker CNPase (2′,3′-Cyclic-nucleotide 3′-phosphodiesterase) (Supplementary Fig. 3h, i) both in the cortex and thalamus. APP also accumulated in CTE patients (Supplementary Fig. 4a, b). Although Aβ plaques (Aβ40 or Aβ42) were detected in some CTE patients (Supplementary Fig. 4c−f), plaque volume between cases and controls did not differ (Fig. 3c, Supplementary Fig. 4c−f).

### *Cis* mAb improves acute phase outcomes after ssTBI

**Fig. 4:**
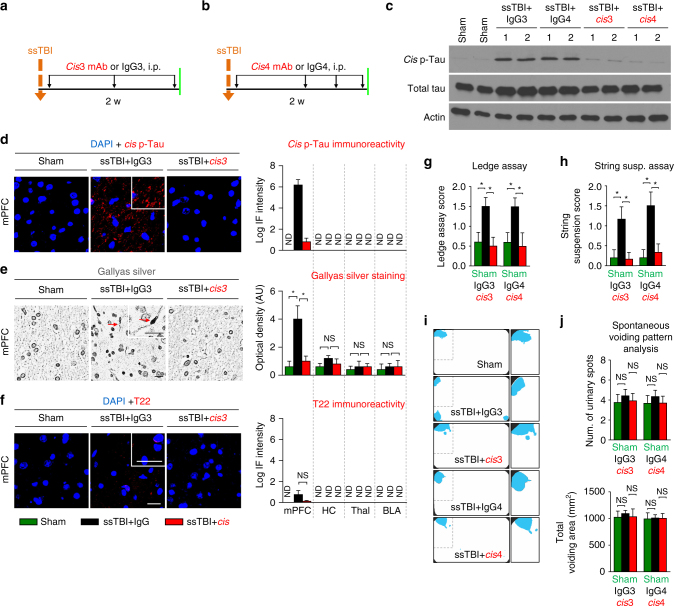
Eliminating *cis* P-tau in ssTBI mice with *cis* mAb prevents a range of pathological and functional outcomes during the acute phases. Mice were subjected to one of two treatment regimens involving 3 or 4 i.p. injections of *cis* mAb for 2-weeks **a**, **b**, followed by functional, biochemical, and pathological examinations. Orange arrows, ssTBI; black arrows, antibody injection; green lines, functional, or pathological assays. *Cis* mAb treatment of ssTBI mice for 10 days effectively eliminated *cis* P-tau induction and total tau accumulation, as detected by immunostaining and immunoblotting **c**, **d**, restored axonal pathology by Gallyas silver staining **e**, without any tau oligomerization **f**, as well as prevented sensorimotor coordination deficits, as detected by Ledge assay **g** and string suspension **h** at 2 weeks after injury, with no change in urinary pattern at this time (that is, all mice urinated in the corner of cages indicated by blue fluorescent urine, as expected for normal mice) **i**, **j**. Microscope images correspond to the medial prefrontal cortex of sham (left), ssTBI + IgG (middle), and ssTBI + *cis* mAb (right) with quantification data in different brain regions being present at right panels. Inset images are the high magnification image of selected area denoted by the white. Scale bar, 40 μm. Axonal bulb also referred to as a retraction ball indicated with red arrow in Gallyas silver staining. mPFC, medial prefrontal cortex; HC, hippocampus; Thal, thalamus; BLA, basolateral amygdala. ND, not detectable; NS, not significant. Brains from 4−5 WT male mice were studied in immunohistochemistry, 5−6 mice underwent urinary pattern test and 9−10 WT mice underwent other behavioral studies per group. The data were presented as means ± SEM. The *p*-values were calculated using unpaired two-tailed parametric Student’s *t*-test. *p < 0.05, **p < 0.01, ***p < 0.001, ****p < 0.0001

**Fig. 5:**
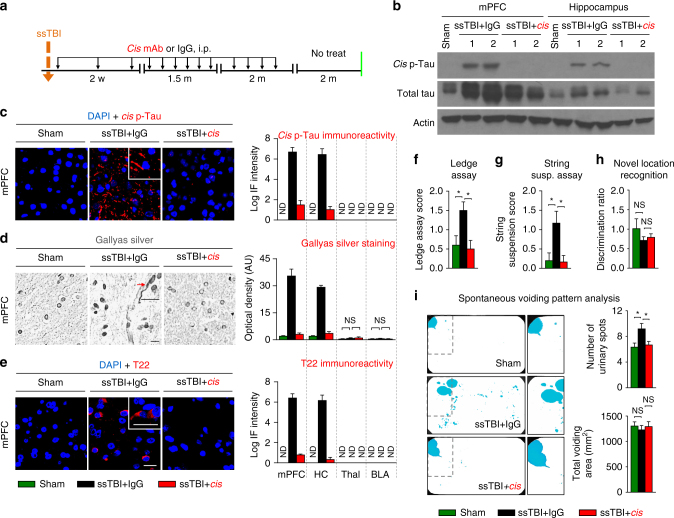
Eliminating *cis* P-tau in ssTBI mice with *cis* mAb prevents a range of pathological and functional outcomes during the chronic phase. Mice were subjected to long-term treatment regimens over 4-months, followed by a 2-month washout **a** and then underwent functional, biochemical, and pathological examinations. Orange arrows, ssTBI; black arrows, antibody injection; green lines, functional, or pathological assays. *Cis* mAb treatment of ssTBI mice for 4 months effectively eliminated *cis* P-tau induction and total tau accumulation **b**, **c**, and prevented the development of axonal pathology **d** and tau oligomerization **e** both in the cortex and hippocampus, as well as prevented sensorimotor coordination deficits, as detected by Ledge assay **f** and string suspension **g** and urinary incontinence as assayed by spontaneous urinary pattern **i** at 6 months after injury, with no deficit in novel object location recognition **h**. Microscope images correspond to the medial prefrontal cortex of sham (left), ssTBI + IgG (middle), and ssTBI + *cis* mAb (right) with quantification data in different brain regions being present at right panels. Inset images are the high magnification image of selected area denoted by the white. Scale bar, 40 μm. Axonal bulb also referred to as a retraction ball indicated with red arrow in Gallyas silver staining. mPFC, medial prefrontal cortex; HC, hippocampus; Thal, thalamus; BLA, basolateral amygdala. ND, not detectable; NS, not significant. Brains from 4−5 WT male mice were studied in immunohistochemistry, 5−6 mice underwent urinary pattern test and 9−10 WT mice underwent other behavioral studies per group. The data were presented as means ± SEM. The *p*-values were calculated using unpaired two-tailed parametric Student’s *t*-test. *p < 0.05, **p < 0.01, ***p < 0.001, ****p < 0.0001

The association of early *cis* P-tau with clinical outcomes after severe TBI, and the correlation of *cis* P-tau with other neurodegenerative consequences suggest that *cis* P-tau may be crucial for the development and progression of not only tau pathology, but also for other short-term and long-term outcomes of ssTBI and rmTBI. To address this question, we subjected young adult C57BL6 mice to a weight drop closed head injury, delivering a 54-gram weight from a height of 60 inches to the cranium of an unrestrained mouse, allowing rapid rotational acceleration of the head to produce single severe/moderate impact closed head TBI (ssTBI). This resulted in nearly 100% convulsive activity and obvious *cis* P-tau induction, without a skull fracture or contusion^38, 48, 59, 60^. After injury, we examined the relationships between *cis* P-tau and various pathological and functional changes at 48 h, 2 weeks and 6 months after injury. As shown previously, we found that *cis*, but not *trans*, P-tau was induced in the cortical axons at 48 h after ssTBI (Supplementary Fig. 5a, b). Importantly, traumatic axonal injury and pathology was obvious at this time, as detected by Gallyas silver-positive axonal bulbs (Supplementary Fig. 5c), and supported by axonal accumulation of APP (Supplementary Fig. 5d). Notably, changes observed soon after injury were limited to the cortex close to the impact site, but not found in deeper brain regions, such as the hippocampus and thalamus (Supplementary Fig. 5). We also investigated whether other tau and/or neurodegenerative pathologies might appear acutely after ssTBI using well-characterized antibodies. We found no clear evidence of tau oligomers, early or late tangles (Supplementary Fig. 5e−g) or other neurodegenerative changes including GFAP-positive astrocyte or Iba1-positive microglia (Supplementary Fig. 5h, i), Aβ (Supplementary Fig. 5j) or TDP-43 pathology (Supplementary Fig. 5k), neuronal loss (as detected by the neuronal specific nuclear protein-NeuN) (Supplementary Fig. 5l) or demyelination (by the oligodendrocyte marker CNPase) (data not shown) at 48 h after injury. At later time points after injury, *cis* P-tau (Fig. 4c, d, Supplementary Fig. 14) and diffuse axonal injury (DAI) (Fig. 4e) persisted, along with the appearance of astrogliosis (Supplementary Fig. 6e) in the cortex at 2 weeks, spreading deeper to other brain regions such as the hippocampus at 6 months (Fig. 5, Supplementary Figs 6, 15). Other tau pathologies, including tau oligomers, early and late tangles, and neurodegenerative pathologies appeared in deeper brain regions such as hippocampus at 6 months (Fig. 5, Supplementary Fig. 6), but not at 2 weeks after ssTBI (Fig. 4, Supplementary Fig. 6). These results suggest that in a severe preclinical impact and acceleration TBI model, closed head injury induces prominent *cis* P-tau along with DAI without any other commonly known tau pathology or other secondary pathologies in the brain surface cortex acutely after injury. With time, *cis* P-tau spreads to deeper brain regions along with the appearance of other tau pathologies and other secondary and neurodegenerative pathologies.

To test whether *cis* P-tau is causative rather than just associated with functional and neurodegenerative features of TBI, we treated ssTBI mice with *cis* P-tau mAb. We have previously shown that peripherally administrated *cis* P-tau mAb is able to enter the brain and can effectively eliminate *cis* P-tau by targeting intracellular *cis* P-tau for proteasome-mediated degradation as well as preventing extracellular *cis* P-tau from spreading to other neurons after TBI in mice^48^. This is consistent with many previous results showing that immunotherapy can effectively remove toxic proteins in the brain^27, 28, 53, 54^. Therefore, we evaluated the effects of *cis* P-tau mAb treatment on other neurodegenerative pathologies and functional outcome. Although there is no common scoring system to assess functional outcomes in preclinical TBI models to correlate with the widely used clinical outcome measure, GOS, we chose functional outcomes based on a robust literature in preclinical TBI models^60–62^. After treating ssTBI mice with *cis* mAb or IgG isotype control for 2 weeks with 3 or 4 intraperitoneal (i.p.) injections (200 µg each) over 10 days (Fig. 4a, b), *cis* mAb eliminated toxic *cis* P-tau induction (Fig. 4c, d, Supplementary Fig. 14), silver-positive inclusion (Fig. 4e), APP accumulation (Supplementary Fig. 6b) and astrogliosis (Supplementary Fig. 6e) in the cortex without changing physiological *trans* P-tau (Supplementary Fig. 6a). Treatment with *cis* P-tau mAb also prevented sensorimotor coordination deficits, as detected by Ledge assay^63^ (Fig. 4g) and string suspension^64^ (Fig. 4h). At 2 weeks after injury, we did not detect significant deficits in novel location recognition memory^65, 66^ (Supplementary Fig. 7k) and spontaneous urinary pattern^67^ (Fig. 4i, j) in the two TBI groups when compared with the sham control. In addition, we did not detect tau oligomerization (Fig. 4f), early and late tangle formation (Supplementary Fig. 6c, d), Iba1-positive microglia (Supplementary Fig. 6f), TDP-43 pathology (Supplementary Fig. 6g), Aβ pathology (Supplementary Fig. 7a, b), neuronal loss (Supplementary Fig. 7c) or demyelination (Supplementary Fig. 7d, e) in the two TBI groups compared with sham mice at 2 weeks after severe injury.

### *Cis* mAb improves chronic phase outcomes after ssTBI

In contrast to 2 weeks after ssTBI, at 6 months the development and spreading of other tau pathology (tau oligomers, early and late tangles) and neurodegenerative pathologies, together with the emergence of more behavioral deficits, were observed (Fig. 5 vs. Fig. 4). Notably, intermittent *cis* mAb treatment for 4 months (Fig. 5a) not only eliminated and blocked spreading of *cis* P-tau (Fig. 5b, c, Supplementary Fig. 15), axonal pathology (Fig. 5d) and astrogliosis (Supplementary Fig. 6l) into the hippocampus without affecting physiologic *trans* P-tau (Supplementary Fig. 6h), but also prevented tau oligomerization (Fig. 5e), tangle-like formation (Supplementary Fig. 6j, k), and APP accumulation (Supplementary Fig. 6i). We did not detect reactive microgliosis (Supplementary Fig. 6m), TDP-43 pathology (Supplementary Fig. 6n), Aβ pathology (Supplementary Fig. 7f, g), neuronal loss (Supplementary Fig. 7h) or demyelination (Supplementary Fig. 7i, j) in all three groups at 6 months after ssTBI. Functionally, ssTBI did not lead to deficits in the novel location recognition (Fig. 5h) or baseline exploratory/locomotion activity assayed by dim-light open field test (Supplementary Fig. 8a−c). However, although *cis* mAb treatment did not improve the performance on the Morris water maze (Supplementary Fig. 8f), as shown previously^48^, it restored performance to sham level for other functional outcomes including sensorimotor and coordination imbalance as detected by the Ledge assay (Fig. 5f, Supplementary Movie 1) and string suspension (Fig. 5g, Supplementary Movie 2), and urinary incontinence, as detected by spontaneous urinary pattern (Fig. 5i). Sensorimotor/coordination defects^68–70^ and urinary incontinence^71, 72^ are major clinical problems in severe TBI patients.

### Delayed *cis* mAb administration improves outcomes after ssTBI

**Fig. 6:**
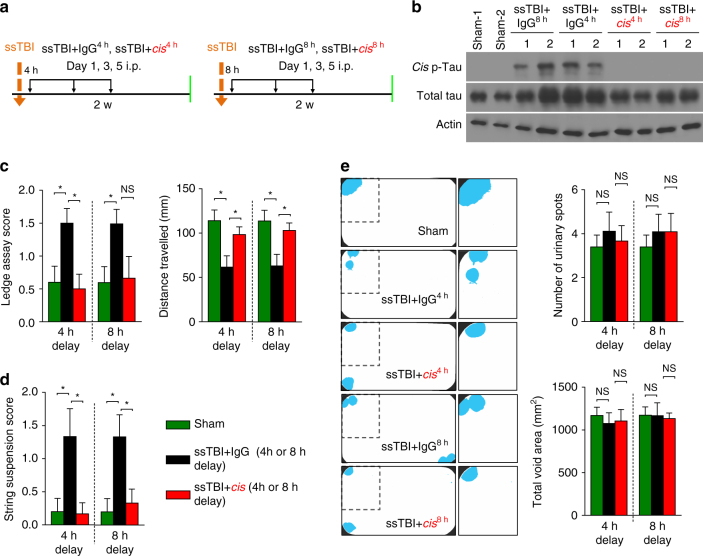
Treating ssTBI mice with 3 i.p. *cis* mAb with 4 or 8 h delay eliminates *cis* P-tau and restores sensorimotor coordination deficits at 2 weeks after injury. Treating ssTBI mice with 3 i.p. of cis mAb even with 4 or 8 h delay **a** effectively eliminated cis P-tau induction **b**, and restored sensorimotor coordination deficits (ledge assay and string suspension assay) **c**, **d**. Urinary incontinence was not observed after 2-weeks **e**. Orange arrows, ssTBI; black arrows, antibody injection; green arrows, functional, or pathological assays. The data were presented as means ± SEM. The *p*-values were calculated using unpaired two-tailed parametric Student’s *t*-test. *p < 0.05, NS, not significant

As a proof of concept to evaluate the efficacy of a shorter course of *cis* mAb treatment with delayed administration, we investigated two shorter treatment regimens consisting of four doses (4 i.p. injections at days 1, 3, 7, and 10 starting immediately after ssTBI injury) and 3 doses (3 i.p. injections at days 1, 3, and 5, starting up to 8 h after ssTBI) (Fig. 6a). The shorter regimens were effective in eliminating *cis* P-tau induction (Fig. 6b, Supplementary Fig. 16) and preventing sensorimotor/coordination imbalance at 2 weeks after ssTBI (Fig. 6c, d), even when *cis* mAb treatment was delayed 4 or 8 h after injury (Fig. 6a). Again, there was no obvious defect between sham and the two treated TBI groups in spontaneous urinary pattern (Fig. 6e). These data suggest that a short-term, intensive loading dose of *cis* mAb treatment, started at delayed time points after injury, might be sufficient to eliminate *cis* P-tau induction in rodent models, but further evaluation of the treatment window and duration of therapy is warranted.

### *Cis* mAb prevents CTE pathology and dysfunction after rmTBI

**Fig. 7:**
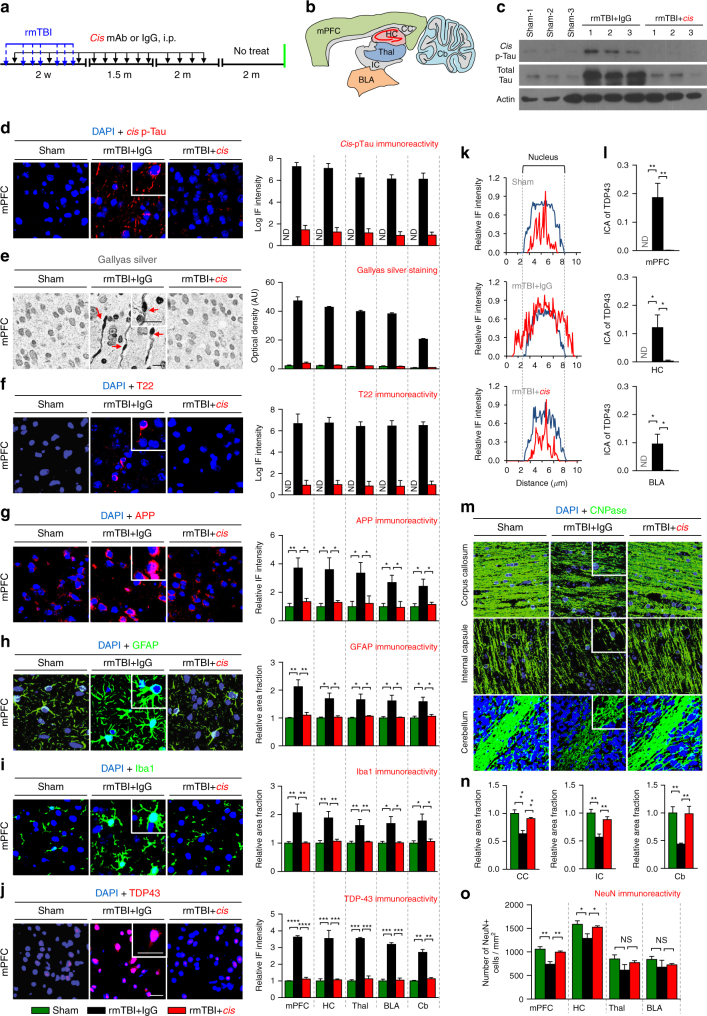
Eliminating *cis* P-tau in rmTBI mice with *cis* mAb prevents the development of a range of pathological features resembling those found in human CTE. Mice were subjected to seven mild TBI events over 9 days and were treated with *cis* mAb or IgG isotype control over 4 months, followed by 2 months of washout, before assaying pathologies in different brain regions **a**, **b**. Blue arrows, rmTBI; black arrows, antibody injection; green line, functional, or pathological assays. *Cis* mAb treatment of rmTBI mice eliminated induction and spreading of *cis* P-tau and total tau **c**, **d**, prevented the development and spreading of axonal pathology **e**, tau oligomerization **f**, APP accumulation **g**, GFAP-positive astrocyte **h**, Iba-positive microglia **i**, TDP-43 pathology with increased cytoplasmic mislocalization of TDP-43 **j**−**l**. Line graphs showing the relative IF intensity of TDP-43 across a single cell **k**, **l** (Blue line, DAPI; red line, TDP-43). *Cis* mAb treatment also prevented the demyelination as detected by CNPase IF **m**, **n**, and neuronal loss **o** in different brain regions. Shorter exposure of ECL for total tau immunoblots in **c** was used due to a huge increase in total tau in rmTBI mice, as expected because *cis* P-tau is resistant to protein degradation. Microscope images corresponded to the medial prefrontal cortex of sham (left), rmTBI + IgG (middle), and rmTBI + *cis* mAb (right) with quantification data in different brain regions being present at right panels. Inset images are the high magnification image of selected area denoted by the white. Scale bar, 40 μm. Red arrows point to axonal bulb in Gallyas silver staining. mPFC, medial prefrontal cortex; HC, hippocampus; Thal, thalamus; BLA, basolateral amygdala; CC, corpus callosum; IC, internal capsule; Cb, cerebellum. ND, not detectable; NS, not significant. Brains from 4−5 WT male mice were studied in immunohistochemistry per group. The data are presented as means ± SEM. The *p*-values were calculated using unpaired two-tailed parametric Student’s *t*-test. *p < 0.05, **p < 0.01, ***p < 0.001, ****p < 0.0001

**Fig. 8:**
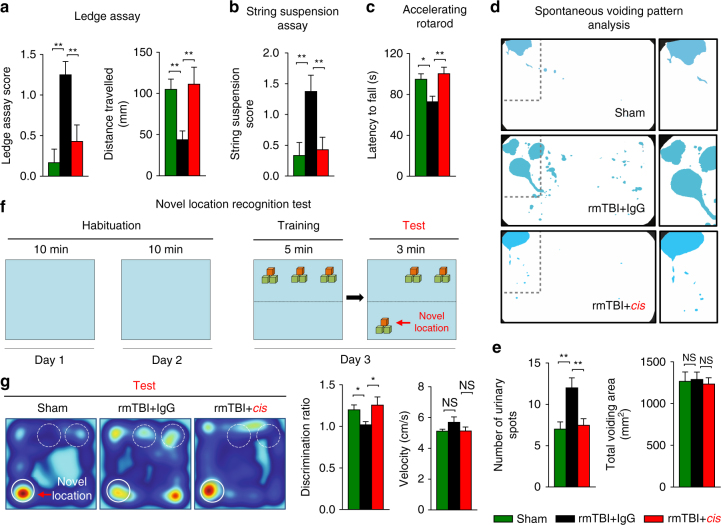
Eliminating *cis* P-tau in rmTBI mice with *cis* mAb prevents the development of clinically relevant functional deficits. *Cis* mAb treatment of rmTBI mice prevents sensorimotor coordination deficits, as detected by Ledge assay **a**, string suspension **b** and accelerated rotarod **c**, and urinary incontinence, as assayed by spontaneous urinary pattern analysis **d**, **e** and memory deficit, as assayed by novel object location recognition test at 6 months after injury **f**, **g**. 5−6 mice underwent urinary pattern test and 9−10 WT mice underwent other behavioral studies per group. The data are presented as means ± SEM. The *p*-values were calculated using unpaired two-tailed parametric Student’s *t*-test. *p < 0.05, **p < 0.01, ***p < 0.001, ****p < 0.0001

We next asked whether *cis* mAb is able to prevent the development of CTE-like pathology using an established weight-drop model of rmTBI^38, 48, 60^. Mice underwent seven injuries in 9 days (54-gram weight, 28″ drop height) and were treated with *cis* mAb or control IgG isotype for 4 month, followed by 2 months of washout without treatment, as described in Methods section. Functional outcomes were assessed at 6 months after injury after which mice were killed and examined for histopathological outcomes (Fig. 7a, b). Compared to ssTBI mice at 6 months, rmTBI mice demonstrated stronger evidence of a range of secondary pathologies, including axonal pathology, *cis* P-tau and tau tangles, Aβ loading, gliosis, neuroinflammation, TDP-43 pathology and demyelination, as well as wider and deeper spreading of pathologies to various brain regions including white matter and cerebellum (Fig. 7, Supplementary Fig. 9), similar to human CTE (Fig. 3, Supplementary Fig. 3). Treatment with *Cis* mAb eliminated the induction of *cis* P-tau (Fig. 7c, d, Supplementary Fig. 17) and axonal pathology (Fig. 7e), and prevented total tau accumulation (Fig. 7c), tau oligomerization (Fig. 7f), and tangle-like formation (Supplementary Fig. 9b, c, Supplementary Fig. 10c, d) as well as neuron loss (Fig. 7o) across different brain regions (Fig. 7b). Furthermore, *cis* mAb also blocked other secondary pathologies after TBI including APP accumulation (Fig. 7g), Iba1-positive microglia and GFAP-positive astrocytes (Fig. 7h, i), TDP-43 pathology (Fig. 7j) with increased cytoplasmic mislocalization of TDP-43 (Fig. 7k, l), and demyelination (Fig. 7m, n, Supplementary Fig. 9f−g) throughout the brain including the cortex, hippocampus, thalamus, amygdala, and even cerebellum. Anti-paired helical filament (PHF)-tau immunostaining and Thioflavin-S staining also showed robust tangle-like pathology in the frontal cortex 6 months after rmTBI (Supplementary Fig. 10c, d), although not so obvious after ssTBI (Supplementary Fig. 10a, b). These tangle-like pathologies were effectively mitigated by elimination and neutralization of *cis* P-tau by treatment with *cis* mAb (Supplementary Fig. 10). Notably, we also observed tangle-like pathology and increased astrocytosis in the periventricular and perivascular elements in rmTBI mice (Supplementary Figs. 11, 12) resembling those found similar in humans with CTE, which were also mitigated by neutralization of *cis* P-tau by treatment with *cis* mAb (Supplementary Figs. 11, 12). rmTBI led to a statistical trend in increased Aβ deposition and treatment with *cis* mAb also prevented the increase (Supplementary Fig. 9d, e). Moreover, although rmTBI did not lead to deficits in exploratory or locomotion activity (Supplementary Fig. 8d, e), *cis* mAb prevented the development of a range of other clinically relevant functional outcomes including sensorimotor coordination imbalance (Fig. 8a−c), urinary incontinence (Fig. 8d, e), and memory deficit as detected by novel object location recognition test (Fig. 8f, g). These results indicate that *cis* mAb treatment eliminates *cis* P-tau induction and spread, and also prevents the development of a range of CTE-like pathological features and functional outcomes after rmTBI.

### Efficacy of *cis* mAb in improving outcomes across studies

To test the efficacy of *cis* mAb to improve functional outcomes across the injury (ssTBI and rmTBI) and treatment (immediate or delayed) regimens, we pooled data and performed two statistical analyses. First, we normalized the data of each experiment by sham for comparing experiments, and then combined all of the data and calculated the mean and SD, followed by calculating combined fold change, *cis* mAb effect size, Cohen’s d or *z*-score effect size, and combined *p*-value. Cohen d, defined as standardized mean difference, is commonly used as an effect size for continuous data following a normal distribution to indicate the standardized difference between two means^73^. Cohen d is scaled and classified as small (*d* = 0.2), medium (*d* = 0.5), large (*d* = 0.8), very large (*d* = 1.2), or huge (*d* = 2.0). For three tests (Ledge assay, string suspension and voiding pattern tests), which do not have continuous data following a normal distribution, we instead calculated the *z*-score effect size from the rank sum test and divided by the square of the number of observations, to get a statistic that may be a nonparametric alternative to Cohen’s d^73^. This data analysis showed that *cis* mAb prevented the development of an array of histopathological and functional outcomes after ssTBI or rmTBI (Table 1). To further support these findings, we employed factor analysis^74^ for the functional and pathological outcomes across all ssTBI and rmTBI studies, under the assumption that treatment addresses a latent behavior construct factor, i.e., a common mechanism, across studies (Supplementary Table 4), as described in Methods section. Factor analysis is a statistical method intended to explain the relationships among several difficult to interpret, correlated variables in terms of a few conceptually meaningful, relatively independent factors and is frequently employed in clinical neuropsychiatric studies^74^. Using conventional factor loading cutoff of 0.3 to determine variable retention^74^, we performed factor analysis for histopathological outcomes (7 histopathological outcomes in cortex and hippocampus), functional outcomes (three behavior assays), and combined histopathological and functional outcomes. In each of these factor analyses, the scree plot demonstrated one factor to be retained for linear regression leaving one latent construct each for histopathological outcomes, functional outcomes, and combined histopathological and functional outcomes. We next performed a distinct linear regression for each latent construct outcome with indicator variables for injury and treatment as the predictors. On linear regression, we found that IgG treated mice were different than sham mice or *cis* mAb treated mice, but there was no difference between sham mice and *cis* mAb treated mice in terms of the latent histopathology, latent behavior or combination constructs (Supplementary Table 4). Both data analyses demonstrate the potent efficacy of *cis* mAb in preventing the development and progression of histopathological and functional outcomes across ssTBI and rmTBI studies.

**Table 1:** Combined therapeutic outcomes of *cis* mAb treatments in ssTBI mice or rmTBI mice regimens

**TBI-related outcomes**	**Experimental tests**	**Treatment regimen (as used in figures)**	**No of total mice used**	**No of mice each group**	**Pathological and functional outcomes (fold ± SD)**				Cohen’s d* **or** ***z*** -score effect size**	***p*** **-value**
					**Combined IgG/Sham**		Combined ***Cis*** **mAb/ Sham**	***Cis*** **mAb combined effect size**		
*Selected pathological outcomes*										
*Cis* P-tau	*Cis* P-tau (cortex)	5a; 4a; 7a	39	3−4	100.7 ± 0.28	11.3 ± 0.25		8.91 ± 0.36	5.99	≤0.0001
	*Cis* P-tau (hippo.)	5a; 4a; 7a	39	3−4	67.8 ± 0.88	5.87 ± 0.19		11.5 ± 0.91	1.98	≤0.0001
*Trans* P-tau	*Trans* P-Tau (cortex)	5a; 4a; 7a	39	3−4	1.00 ± 0.22	1.00 ± 0.21		1.00 ± 0.23	0.02	>0.05
	*Trans* P-Tau (hippo.)	5a; 4a; 7a	39	3−4	1.04 ± 0.24	1.03 ± 0.26		1.01 ± 0.21	0.03	>0.05
Axonal injury	Gallyas silver (cortex)	5a; 4a; 7a	39	3−4	20.8 ± 6.51	1.66 ± 0.58		12.5 ± 6.51	2.26	≤0.0001
	Gallyas silver (hippo.)	5a; 4a; 7a	39	3−4	15.2 ± 4.65	1.46 ± 0.56		10.5 ± 4.67	2.21	≤0.0001
Other tau pathology	T22 (cortex)	5a; 4a; 7a	39	3−4	81.8 ± 0.81	8.98 ± 0.22		9.12 ± 0.83	2.09	≤0.0001
	T22 (hippocampus)	5a; 4a; 7a	39	3−4	64.4 ± 0.85	5.85 ± 0.21		11.0 ± 0.87	1.93	≤0.001
	AT8 (cortex)	5a; 4a; 7a	39	3−4	69.0 ± 0.84	12.3 ± 0.27		5.63 ± 0.89	1.75	≤0.0001
	AT8 (hippocampus)	5a; 4a; 7a	39	3−4	63.8 ± 0.81	7.16 ± 0.22		8.91 ± 0.87	1.85	≤0.0002
	AT100 (cortex)	5a; 4a; 7a	39	3−4	59.8 ± 0.84	7.39 ± 0.22		8.08 ± 0.86	1.71	≤0.0001
	AT100 (hippocampus)	5a; 4a; 7a	39	3−4	60.2 ± 0.80	6.56 ± 0.21		9.18 ± 0.81	1.86	≤0.0003
APP accumulation	APP (cortex)	5a; 4a; 7a	39	3−4	3.82 ± 0.43	1.39 ± 0.19		2.74 ± 0.46	2.09	≤0.0001
	APP (hippocampus)	5a; 4a; 7a	39	3−4	2.59 ± 0.46	1.12 ± 0.12		2.30 ± 0.45	1.47	≤0.006
Neuron inflammation	GFAP (cortex)	5a; 4a; 7a	39	3−4	1.62 ± 0.13	1.06 ± 0.03		1.52 ± 0.14	1.68	≤0.0005
	GFAP (hippocampus)	5a; 4a; 7a	39	3−4	1.42 ± 0.11	1.03 ± 0.02		1.37 ± 0.11	1.51	≤0.001
*Selected functional outcomes*										
Sensorimotor coordination defects	Ledge test	4a, b; 5a; 6a, b; 7a	99	5−9	2.92 ± 0.38	1.06 ± 0.19		2.74 ± 0.49	0.64**	≤0.0001
	String suspension	4a, b; 5a; 6a, b; 7a	99	5−9	3.32 ± 0.66	1.00 ± 0.24		3.34 ± 0.81	0.56**	≤0.0001
	Accelerating rotarod	7a	27	9	1.22 ± 0.05	0.90 ± 0.03		1.35 ± 0.04	1.22	≤0.03
Cognitive loss	Novel location recog.	7a	27	9	1.27 ± 0.05	0.91 ± 0.03		1.38 ± 0.05	1.21	≤0.03
Urinary control	Voiding pattern	5a; 7a	42	5-9	1.45 ± 0.09	0.93 ± 0.06		1.56 ± 0.10	0.55**	≤0.002

*Cohen d is classified as small (*d* = 0.2), medium (*d* = 0.5), large (*d* = 0.8), very large (*d* = 1.2), or huge (*d* = 2.0). **z-score is a nonparametric alternative to Cohen’s d

## Discussion

Here we demonstrate the significance of *cis* P-tau across a spectrum of TBI mechanisms and pathologic outcomes at acute and chronic time points. Having previously identified *cis* P-tau as an early driver of tau pathology and neurodegeneration after severe closed head TBI in preclinical models and offering a potential link between TBI and neurodegeneration^48–50^, we now demonstrate the relevance of *cis* P-tau to human TBI, including severe single TBI and CTE. In addition, we also define the role of *cis* P-tau in the development and treatment of other short-term and long-term consequences of TBI, including a wide array of CTE-like neurodegenerative features, such as axonal pathology, tau, APP, and TDP-43 pathologies, neuroinflammation, neuronal loss, white matter degeneration and cerebellar pathology, as well as clinically relevant functional deficits, including sensorimotor coordination imbalance, urinary incontinence, and cognitive impairment. Despite a growing clinical literature demonstrating that TBI is an important environmental risk factor for neurodegenerative disease such as CTE^7–10^ and AD^11–14^, the causal link and underlying mechanisms between TBI and these neurodegenerative outcomes remains unclear^8–10^ and the role of tau pathology, a common feature of these neurodegenerative outcomes, is not known^7–10, 19^.

To detect *cis* P-tau acutely after TBI and determine its significance in acute and chronic TBI in humans, we examined *cis* P-tau in autopsy specimens from patients with fatal TBI and CTE, as well as in CSF samples from patients with severe TBI. We found that diverse mechanisms of severe TBI due to motor vehicle accidents, assaults or falls result in acute and robust induction of *cis* P-tau in axons, along with axonal injury mainly in the cortex, but without other neurodegenerative changes in tau, Aβ or TDP-43-related pathologies and Iba1-positive reactive microglia within the first month after injury. We found that *cis* P-tau in the CSF of TBI patients displays dose-dependent neurotoxicity in vitro, and is highly correlated with the clinical outcome of patients with TBI at 1 year after injury, further supporting its pathological significance. However, in human subjects with exposure to repetitive head trauma who are diagnosed with CTE at autopsy, robust *cis* P-tau is not only detected in the brain surface cortex, but also in deeper brain regions such as the thalamus, and is closely associated with a range of neuropathological features of CTE including axonal pathology, tau, APP, and TDP-43 pathologies, neuroinflammation, neuronal loss, white matter degeneration, and cerebellar pathology. These results not only support our previous findings that *cis* P-tau is crucial for the development and progression of tau pathology^48^, but also suggest that *cis* P-tau may be involved in the development and progression of other short-term and long-term outcomes of ssTBI and rmTBI. Although the correlation of *cis* P-tau with the 1 year clinical outcome and pathological changes in human TBI patients is intriguing, large scale longitudinal studies are needed to validate whether *cis* P-tau is a predictive biomarker of injury and recovery. Moreover, there may be additional potential confounding or effect-modifiers, making it difficult to establish a causative role for *cis* P-tau in the development of acute and chronic pathological changes after TBI.

To test whether *cis* P-tau is an early, key mediator of diverse neurodegenerative changes and functional impairment after TBI, we utilized established closed head injury models of ssTBI and rmTBI to evaluate the effects of *cis* mAb therapy on pathological and functional outcomes after injury. Here we demonstrate that ssTBI acutely induces prominent *cis* P-tau before tau oligomerization or tangle formation, or other secondary pathologies in the injured cortex. With time, *cis* P-tau spreads to deeper brain regions along with the appearance of other tau pathology, other secondary and neurodegenerative pathologies as well as functional deficits. Importantly, treating ssTBI mice with *cis* mAb effectively eliminates *cis* P-tau induction, axonal pathology and astrogliosis, and also prevents sensorimotor coordination deficits at 2 weeks after injury. At 6 months after injury, *cis* mAb not only eliminates and blocks spreading of *cis* P-tau, axonal pathology and astrogliosis into the hippocampus, but also prevents other mechanisms of secondary and neurodegenerative pathologies. These include tau oligomerization, tangle formation, gliosis and APP accumulation, as well as prevention of sensorimotor coordination deficits and urinary incontinence.

**Fig. 9:**
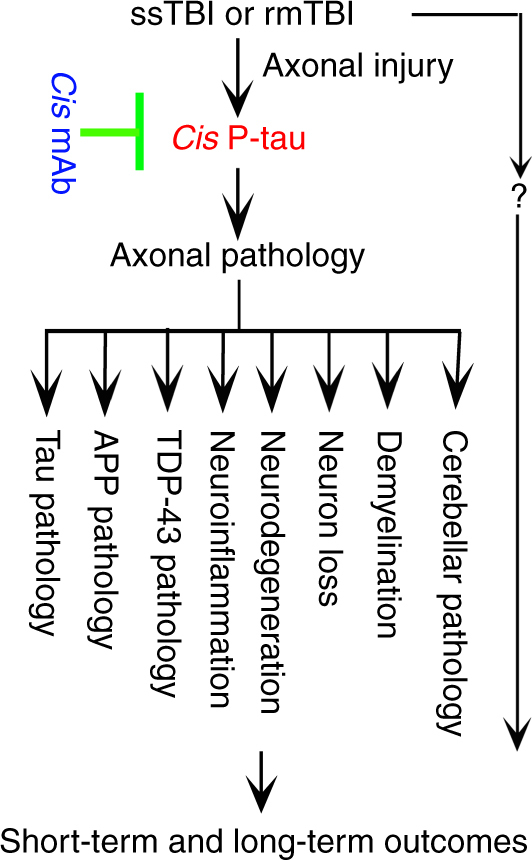
A model for the roles of *cis* P-tau and its mAb in the development and treatment of ssTBI and rmTBI. ssTBI or rmTBI causes persistent and robust *cis* P-tau induction before other tau pathology likely due to axon injury. *Cis* P-tau mainly localizes to axons and causes and spreads axonal pathology, contributing to the development and progression of a range of neuropathological and functional outcomes during acute and chronic phases, including those pathological features resembling human CTE. Treatment of ssTBI or rmTBI mice with *cis* mAb not only eliminates *cis* P-tau and blocks its spreading, but also prevents the development and progression of a range of neuropathological and functional outcomes after injury

We have provided direct evidence that rmTBI in mice is sufficient to induce a wide range of neuropathological features resembling those in human CTE, including axonal pathology, tau, APP, and TDP-43 pathologies, neuroinflammation, neuronal loss, white matter degeneration and cerebellar pathology, as well as clinically relevant functional deficits, including sensorimotor coordination imbalance, urinary incontinence, and cognitive deficit. More importantly, these neuropathological features and functional deficits are almost fully mitigated by elimination and neutralization of *cis* P-tau by treatment with *cis* mAb after rmTBI. These results have not only confirmed our early findings that *cis* P-tau is a precursor of tau pathology and an early driver of neurodegeneration^48–50^, but also suggest that early induction of *cis* P-tau is critical for the development of a range of other pathological and functional outcome after severe or repetitive TBI (Fig. 9). Interestingly, most of these CTE-like pathologies are also found in human AD brains except at different brain regions and Aβ deposition^19, 20^. Previous studies have shown an increase in Aβ deposition in ~50% of patients with CTE^75^ and after acute TBI in humans^76^ though few murine TBI models have demonstrated Aβ deposition after TBI, aside from transgenic mice^77^. We did not observe an increase in Aβ deposition, although Aβ deposition was found in some human CTE brains and some mouse TBI brains, especially 6 months after rmTBI in our studies. Given that Aβ deposition is age-dependent and found normally in many aged brains, the relative paucity of Aβ plaques in our clinical and preclinical studies could reflect the relatively young age of our subjects, <65 years old for the clinical studies and <9 months for the mouse models.

Though Aβ deposition was not a pathologic feature of our TBI models, axonal injury was consistent in both ssTBI and rmTBI models, consistent with human TBI^55^, and treatment with *cis* mAb prevented the progression from traumatic axonal injury to the chronic axonal pathology. Traumatic axonal injury has emerged as one of the most common and important pathological features of closed head injury^55^. It is recognized to cause disruption in axonal transport, followed by secondary disconnection and finally Wallerian degeneration^55, 78, 79^. Although this process was thought to be limited to the acute and sub-acute periods, it has recently been implicated in the development of Alzheimer-like pathologies both in the acute and chronic phrases after TBI^55, 79, 80^. However, molecular mechanisms that mediate traumatic axonal injury to axonal pathology remain elusive^55^. We have previously shown that ssTBI or rmTBI dose-dependently induces *cis* P-tau notably in axons within hours after injury, which disrupts the microtubule network and mitochondrial transport in the axon^48^. Importantly, *cis* mAb treatment not only eliminates axonal *cis* P-tau induction and restores axonal pathologies, including defective microtubules, organelle transport and long-term potentiation, but also prevents the development of a range of short-term and long-term pathological and functional outcomes after ssTBI or rmTBI, as shown here or previously^48^. Moreover, *cis* P-tau is robustly induced, notably in axons, together with traumatic axonal injury hours after closed head injury in humans and mouse models without other secondary pathologies. Others have shown that tau knockout inhibits axonal pathology after rmTBI^52^. Taken together, these results not only further support a major role of traumatic axonal injury in TBI pathologies, but also suggest that *cis* P-tau might mediate traumatic axonal injury to axonal pathology, thereby contributing to the development of many other neuropathological and functional outcomes after TBI (Fig. 9).

Taken together, our data suggest that *cis* p-tau is a possible diagnostic and therapeutic target for immunotherapy. Prior studies have demonstrated that immunotherapy can effectively remove toxic proteins in the brain^27, 28, 53, 54^, with a recent investigation showing the efficacy of peripheral administration of the monoclonal antibody aducanumab in entering into the brain and reducing beta amyloid plaques in clinical trials^54^. Tauopathy, which has previously been implicated in the chronic pathology of TBI and other neurodegenerative diseases, may also be a target for immunotherapy^27, 28^, though tau pathology was not previously identified at early time points after TBI. Here, we have shown that *cis* p-tau appears early after TBI and that CSF *cis* P-tau levels are tightly correlated with clinical outcome after injury. These results are consistent with the previous findings that tau phosphorylated on Thr231 in the CSF is an AD biomarker, correlating with memory loss and predicting AD progression from mild cognitive impairment^81^. Moreover, we have demonstrated that treating ssTBI or rmTBI mice with *cis* mAb is highly effective in preventing short-term and long-term outcomes of TBI in a range of clinically relevant histopathological and functional outcomes. There results offer not only a novel disease mechanism for TBI outcomes but also potential novel targeted therapy for mitigating the short-term or long-term consequences of ssTBI and rmTBI.

## Methods

### Human brain and CSF specimens

Discarded fixed human brain tissues from different brain regions of individuals with acute TBI (Supplementary Table 1) were provided by Dr Colin Smith at the Department of Academic Neuropathology, University of Edinburgh, Little France, UK^32^. Discarded CSF specimens from acute TBI patients (Supplementary Table 2) were obtained from the tissue bank at Boston Children’s Hospital by Rebekah Mannix, who collected human samples originally from Dr. William Gormley at the Department of Neurosurgery, Brigham and Women’s Hospital, Harvard Medical School, Boston, MA, and Dr David O. Okonkwo at the Department of Neurosurgery, University of Pittsburgh Medical Center, Pittsburgh, PA^82^. Discarded fixed human brain tissues from different brain regions of individuals with neuropathologically verified CTE (Supplementary Table 3) were provided by Dr Julian Bailes at the Department of Neurosurgery, NorthShore University Health System at University of Chicago Pritzker School of Medicine^83^, and also by Dr Ann Mckee at the VA-BU-SLI Brain Bank of the Boston University Alzheimer’s Disease Center CTE Program as described previously^7, 48^. Informed consent was obtained from all subjects by respective institutes. Institutional review board approval for tissue donation and our studies on discarded human samples was obtained through the Beth Israel Deaconess Medical Center, the University of Edinburgh, Brigham, and Women’s Hospital, the University of Pittsburgh and Boston Children’s Hospital.

### Immunoblotting analysis and immunodepletion experiments

Immunoblotting analysis and immunodepletion were carried out as described^48, 49^. Briefly, brain tissues or culture cells were lysed in RIPA buffer (50 mM Tris-HCl, pH 7.4, 150 mM NaCl, 2 mM EDTA, 1% NP 40, 0.1% SDS, 0.5% Na-deoxycholate, 50 mM NaF) containing proteinase inhibitors and then mixed with SDS sample buffer and loaded onto a gel after boiling. The proteins were resolved by polyacrylamide gel electrophoresis and transferred to PVDF membrane. After blocking with 5% milk in TBST (10 mM Tris-HCl pH 7.6, 150 mM NaCl, 0.1% Tween 20) for 1 h, the membrane was incubated with primary antibodies (*cis* and *trans* mAbs)^48^, Tau5 (Biosource Camarillo, CA), tubulin (Sigma, St. Louis, MO), and actin antibodies (Sigma, St Louis, MO) in 5% milk in TBST overnight at 4 °C. Then, the membranes were incubated with HRP-conjugated secondary antibodies in 5% milk in TBST. The signals were detected using chemiluminescence reagent (Perkin Elmer, San Jose, CA). The membranes were washed four times with TBST after each step. To deplete *cis* or *trans* p-tau from CSFs, samples were mixed with the *cis* or *trans* mAb antibody at 425 µg/ml in a buffer containing proteinase inhibitors for 3 h at 4 °C and then mixed with protein A/G sepharose for 1 h at 4 °C. The supernatants were collected and added to cell culture application. Immunoblotting results were quantified using Quantity One from BioRad.

### Immunostaining analysis

Immunostaining analysis was carried out as described^48, 49^. The primary antibodies used were *cis* mAb (clone #113) and *trans* mAb (clone #25)^48^, tau tangle-related mAbs AT180, AT8, AT100 (all from Innogenetics, Alpharetta, GA), oligomeric tau T22 polyclonal antibodies (EMD Millipore, Billerica, MA), anti-tau rabbit mAb (E178, Abcam), anti-PHF-1 (ab109390, Abcam), Von Willerbrand factor-vWF (A0082, DAKO), anti-neurofilament mouse mAb (SMI-312, IgG1, Abcam) for labeling axons, anti-MAP2 mAb (SMI-52, IgG1, Abcam) for labeling dendrites, CNPase monoclonal (11-5B) antibody (Abcam) for labeling myelin, TDP-43 polyclonal antibody (proteintech), Iba1 polyclonal antibody (Wako) for microglia, GFAP polyclonal antibody (BioGenex) for astrocytes, APP A4 monoclonal (66-81) antibody (Millipore), anti-Beta-Amyloid (1−42) polyclonal Antibody (Millipore), anti-Beta-Amyloid (1−40) polyclonal Antibody (Sigma-Aldrich) and anti-NeuN AF488-conjugated monoclonal antibody (Millipore) for labeling neurons. Immunofluorescence staining of mouse and human brains was done essentially as described^41, 42, 49^. After treatment with 0.3 % hydrogen peroxide, slides were briefly boiled in 10 mM sodium citrate, pH 6.0, for antigen enhancement. The sections were incubated with primary antibodies overnight at 4 °C. Then, biotin-conjugated secondary antibodies (Jackson ImmunoResearch), streptavidin-conjugated HRP (Invitrogen) were used to enhance the signals. For double immunofluorescence staining, the sections were incubated with Alexa Fluor 488 or 568 conjugated isotype-specific secondary antibodies (Jackson ImmunoResearch, West Grove, PA) for 1 h at room temperature. Manufacturer-supplied blocking buffer (Invitrogen) was used for each reaction. The sections were washed four times with TBS after each step. Labeled sections were visualized with a Zeiss confocal microscope. The gain of confocal laser was set at the level where there are no fluorescence signals including autofluorescence in sections without primary antibody but with secondary antibody. Immunostaining images and their co-localization were quantified using Volocity 6.3 from Perkin Elmer and Fiji/ImageJ Coloc 2, respectively, as described^48, 49^.

### Direct ELISA assay

*Cis* P-tau levels in CSFs were assayed using direct ELISA assay. Human CSF samples were first coated onto ELISA plates. After blocking with buffer containing 1% gelatin in Tris-buffered saline and 0.05% Tween 20, *cis* P-tau was detected with the primary antibody *cis* P-tau mAb at 1:1000 dilution, followed by incubation with biotin-conjugated anti-mouse IgG2b secondary antibody in 0.25–0.5% gelatin in Tris-buffered saline and 0.05% Tween 20 for 1 h and then by streptavidin protein that is covalently conjugated to poly-horseradish peroxidase (HRP) enzyme. The ELISA plates were extensively washed six times with the same buffer after each step. The signals were detected by incubating with TMB substrate solution and measured by Wallac 1420 software at 450 nm, as described^48, 49^.

### Gallyas silver staining

Sections (10 μm thick) of paraformaldehyde-fixed and paraffin-embedded tissues were deparaffinized and then received Gallyas silver stain (reagents from FD NeuroTechnologies), followed by wash in tab water for 5-min and dehydration through a graded series of EtOH (70%, 90%, 100%), for 5-min each, and then clear slides in two changes of xylene solutions. Sections were then covered with mounting media and cover slipped. The Optical Density was measured using with Fiji/ImageJ Coloc 2.

### Thioflavin-S staining

After treatment with 0.3% hydrogen peroxide for 30-min, slides were incubated in 1% thioflavin-S (Sigma-Aldrich, St. Louis, MO, USA) for 15-min at room temperature followed by dehydration through an ethanol series (70%, 90%, and 100%) for 5-min each and two 5-min washes in dH_2_O. Sections were then covered with mounting media and cover slipped.

### Luxol-fast blue staining

After hydration with 95% alcohol for 5-min, slides were incubated in Luxol-fast blue solution (FD NeuroTechnologies) for overnight at 60 °C followed by washed lithium carbonate solution for 5-min at room temperature and two 10-min washes in 70% ethanol. Sections were then rinsed with dH_2_O and covered with mounting media and cover slipped. The Optical Density was measured using with Fiji/ImageJ Coloc 2.

### Cell culture

Neuronal cell lines including SH-SY5Y cells were cultured in Dulbecco’s modified Eagle’s medium (DMEM) containing 10% fetal calf serum. The media were supplemented with 100 Units/ml penicillin/steptomycin. Cell viabilities were examined using Live and Dead cell assay kit (Abcam). After staining cells with the Live and Dead Dye diluted to 5X concentration in PBS, cells were incubated for 10-min at room temperature in the dark. Immunostaining images were then quantified using Fiji/ImageJ Coloc 2.

### Traumatic brain injury

Male C57BL/6 mice (2−3 months old) obtained from the Jackson Laboratories (Bar Harbor, ME) were randomized to undergo injury or sham-injury. The mice were anesthetized for 45 s using 4% isoflurane in a 70:30 mixture of air:oxygen. Anesthetized mice were placed on a delicate task wiper (Kimwipe, Kimberly-Clark, Irving, TX) and positioned such that the head was placed directly under a hollow guide tube. Mouse’s tail was grasped. A 54-gram metal bolt was used to deliver an impact to the dorsal aspect of the skull, resulting in a rotational acceleration of the head through the Kimwipe. Mice underwent single severe injury (ssTBI, 60-inch height) or repetitive mild injuries (rmTBI, seven injuries in 9 days)^38, 48, 59, 84^. Sham-injured mice underwent anesthesia but not concussive injury. All mice were recovered in room air. Anesthesia exposure for each mouse was strictly controlled to 45 s. Briefly, anesthetized young adult wild-type C57BL/6 male mice were exposed to a severe or mild hit or sham hit, removed from the apparatus, monitored until recovery of gross locomotor function, and then transferred to their home cage. Maximum burst pressure compatible with 100% survival and no gross motor abnormalities were ascertained empirically. All these and following animal experiments were approved by the Boston Children’s Hospital, Beth Israel Deaconess Medical Center and/or Boston University and IACUC and complied with the NIH Guide for the Care and Use of Laboratory Animals.

### Antibody treatment of mice

C57BL/6 male mice (2−3 months old) undergoing TBI were randomized to treatment with *cis* p-tau monoclonal mouse antibody (clone #113) or mouse IgG2b in a double-blind manner, as described^48^ with the following modifications. For ssTBI, mice received 3 or 4 doses of *cis* antibody or IgG2b intraperitoneal treatment (200 µg) after injury over 10 days, as described in the text, followed by analysis at 2 weeks after injury or by further treatment 200 µg i.p. weekly for another 1.5 month and biweekly for another 2 months (with total 4 months of treatment) before analyses at 6 months, as described^41, 42, 48, 49^. For rmTBI, mice received intraperitoneal treatments (200 µg) on days 1, 7, 14, and 21, followed by twice a month for 3 months (with total 4 months of treatment) before analyses at 6 months, as described^41, 42, 48, 49^. For all behavioral tests, experimenters were blinded to injury and treatment status, using color-coding stored in a password-protected computer.

### Spontaneous voiding assay

Group housed animals (4–6 per cage) were placed in a clean, empty cage lined with precut 3 MM acid-hardened filter paper (Waltham, MA) with one animal per cage. Voiding assays were conducted over 4 h per day for three consecutive days during which time mice had access to food but not water, as previously described^67^. Filter papers were imaged using UV light and analyzed using Image J Software using the threshold technique in double-blind manner. Image J particle analysis was performed on spots greater than 6 mm^2^ (corresponding to 0.6 μl urine), reducing non-specific marks potentially deposited by paws and tails that pass through urine spots.

### Ledge assay

In the ledge test, mice were placed on the elevated cage’s ledge at a height of 35 cm and 0.8 cm wide, and monitored their movement. Each mouse was tested three times (each test takes 20 s) and scored from 0 to 3 depending on the severity of deficits in a double-blind manner. Scoring is as follows: if the mouse walked along the ledge, without foot faults (i.e., loosing footing) and back into the cage delicately, score of 0; if the mouse demonstrated any foot fault while walking on the ledge, score of 1; if the mouse did not effectively walk on or dismounted the ledge immediately, score of 2; if the mouse fell off the ledge or avoided walking, score of 3.

### String suspension assay

The mouse was permitted to grasp a string only by its forepaws suspended 35 cm above the surface and were then released. Each mouse was tested three times (each test takes 20 s) and scored from 0 to 3 depending on the severity of deficits in double-blind manner. If the mouse was unable to remain on string, score was 3; if it hung by both forepaws and attempted to climb onto the string, score was 2; if both forepaws and one or both hindpaws were around string, score was 1; if four paws and tail were around string, with lateral movement; score was 0.

### Accelerating rotarod test

The mice were placed in the rotating cylinder 4 times per day for two consecutive days totally. Each trial last a maximum of 10 min, during which time the rotating rod accelerated from 4 to 40 r.p.m. over first 5 min of the trial and then remained at the maximum speed for the remaining 5 min. Animal were rested at least 10 min between trials to avoid fatigue and exhaustion.

### Dim-light open field test

Mice were placed in the center of a brightly lit (30−50 lux) chamber of the open field apparatus (40 cm diameter). Movements of the animals were tracked by an automatic monitoring system (Noldus Ethovision XT) for 20 min. Horizontal motor (distance traveled) and central activity (distance traveled in center/total distance traveled) were evaluated.

### Morris water maze

A Morris water maze (MWM) paradigm was used to evaluate spatial learning and memory. The apparatus consisted of a white pool (83 cm diameter, 60 cm deep) with water filled to 29 cm depth, at ~24 °C. Intra-maze and extra-maze cues were included. The target (a round, clear, plastic platform 10 cm in diameter) was placed 1 cm below the surface of the water. During hidden and visible platform trials, mice were randomized to one of four starting quadrants. Mice were placed in the tank facing the wall and allocated 80 s to find the platform, mount the platform, and remain on it for 5 s. Mice were then dried under a heat lamp before their next run. The time until the mouse mounted the platform (escape latency) was recorded. Mice that did not mount the platform in the allocated 80 s were guided to the platform by the experimenter and allowed 10 s to become acquainted with its location. A maximum of two trials per mouse were carried out per day, each trial consisting of four runs, with a 45-minute break between trials (acquisition). For visible platform trials (vision), a red reflector was used to mark the top of the target platform. For probe trials, mice were placed in the tank with the platform removed and given 60 s to explore the tank. Noldus Ethovision 9 software tracked swim speed, total distance moved, and time spent in the target quadrant where the platform was previously located.

### Novel location recognition test

The Novel object recognition test consisted of an open field-box (44 × 44 cm). The habituation period was 5 min daily of free exploration in the arena containing two different objects (15 mm diameter) over 3 days. On test day, the animals were allowed to explore three identical objects (with same color Lego sets) placed into the area in fixed location for 6 min and the time spent inspecting the individual objects was recorded (Noldus Ethovision XT). Without any time-interval the animals were replaced into the box where one object was placed into a new location the mice were allowed to explore them for an additional 3 min. The floor was covered with sawdust (1 cm deep, used and saturated with the odor of the animals) during habituation and test trials. The discrimination ratio for the novel location of the object in was analyzed as previously described^65, 66^.

### Data acquisition and statistical analysis

We estimated the sample size considering the variation and mean of the samples. All surviving animals or samples were included in the analyses except a few mice died immediately after brain injury. Animals were randomly assigned groups for in vivo studies and for mAb treatment experiments, group allocation and outcome assessment were also done in a double-blinded manner. For all behavioral and histopathological tests, experimenters were blinded to injury and treatment status, using color-coding stored in a password-protected computer. Data acquisition and analysis obtained in an unbiased fashion. All data are presented as the means ± s.d. or s.e.m, followed by determining significant differences using the two-tailed Student’s *t*-test for quantitative variables or ANOVA test for continuous or three or more independent variables or one-way ANOVA with Bonferroni posthoc test, and significant *p*-values < 0.05 are shown. In addition, to evaluate for a global effect across behavior tests and TBI treatment groups, we subjected behavioral performance measures to a factor analysis^74^. Factor analysis is a statistical method intended to explain the relationships among several difficult to interpret, correlated variables in terms of a few conceptually meaningful, relatively independent factors and is frequently employed in clinical neuropsychiatric studies. The factors are not measured directly but are inferred from the variables that represent the factor^74^. We determined the number of factors to be retained based on their eigenvalues and by the change in slope of the scree plot. We used a conventional factor loading cutoff of 0.3 to determine variable retention. Variables that did not load on any of the rotated factors were removed and the factor analysis was repeated to produce the final factor solution, which included many histopathological outcomes and/or three functional outcomes (Supplementary Table 2). We then calculated factor scores for each retained factor. Finally, we developed a regression model with the factor score as the dependent variable the treatment group as the predictor, to obtain an overall *p*-value for the effect of treatment on the behavioral performance measure.

### Data availability

All data generated or analyzed during this study are included in this published article (and its Supplementary Information) or from the corresponding author upon reasonable request.

## Electronic supplementary material

Supplementary information

Supplementary Movie 1

Supplementary Movie 2
