# Supplementary material for: Cis P-tau is induced in clinical and preclinical brain injury and contributes to post-injury sequelae
Source: Nat Commun. 2017 Oct 17;8:1000. doi: 10.1038/s41467-017-01068-4 (PMC5645414; doi:10.1038/s41467-017-01068-4)
Supplement: Supplementary file 1 — Supplementary information [file 41467_2017_1068_MOESM1_ESM.pdf]

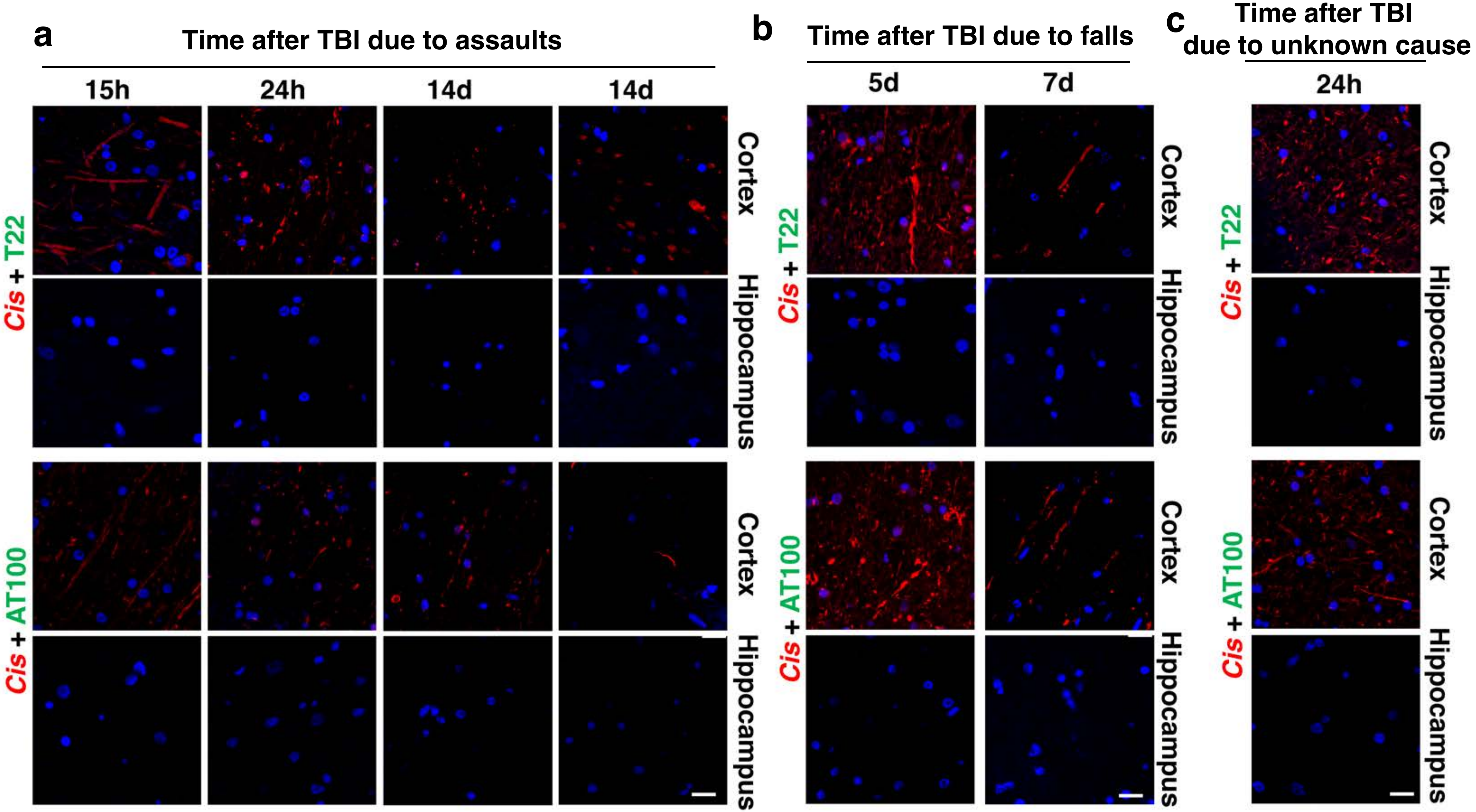

Supplementary Figure 1. Severe TBI in humans due to assaults or falls acutely and robustly induces cis P-tau in the absence of tau oligomerization and tangle formation. Cortical sections of severe human TBI due to assaults (a), falls (b) or an unknown cause (c) were doubly immunostained with cis mAb (red) and T22 (tau oligomers) or AT100 (tau tangles), followed by confocal microscopy. Normal controls as well as CTE and AD brains were used as negative and positive controls, respectively.

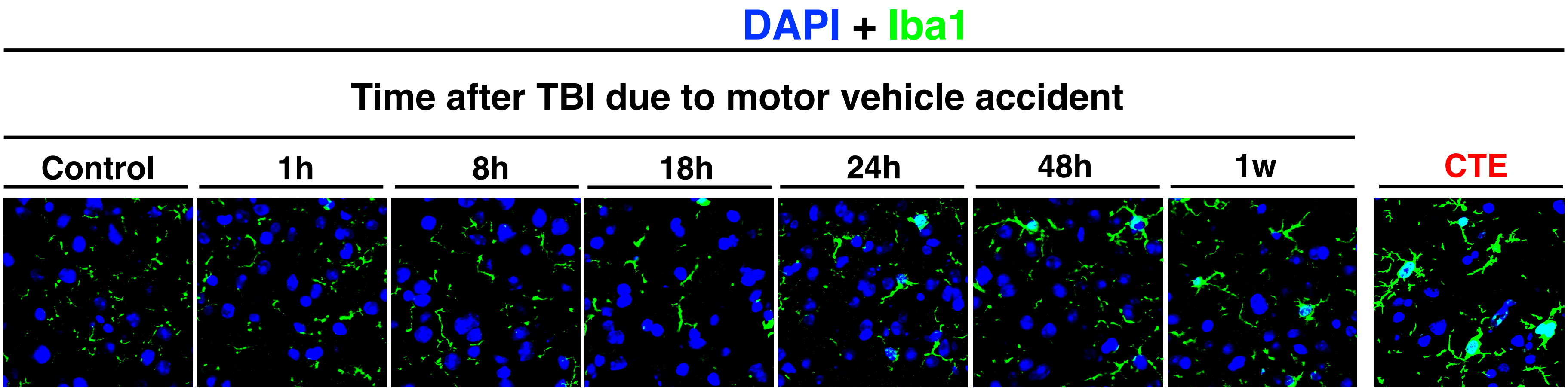

Supplementary Figure 2: Severe TBI in humans due to motor vehicle accidents at different survival times acutely induces tendency towards increased Iba1 staining intensity in the cortex with increasing survival time. Cortical sections of severe human TBI due to motor vehicle accidents at different survival times were immunostained with Iba1 (microglia), followed by confocal microscopy. Normal controls as well as CTE brains were used as negative and positive controls, respectively.

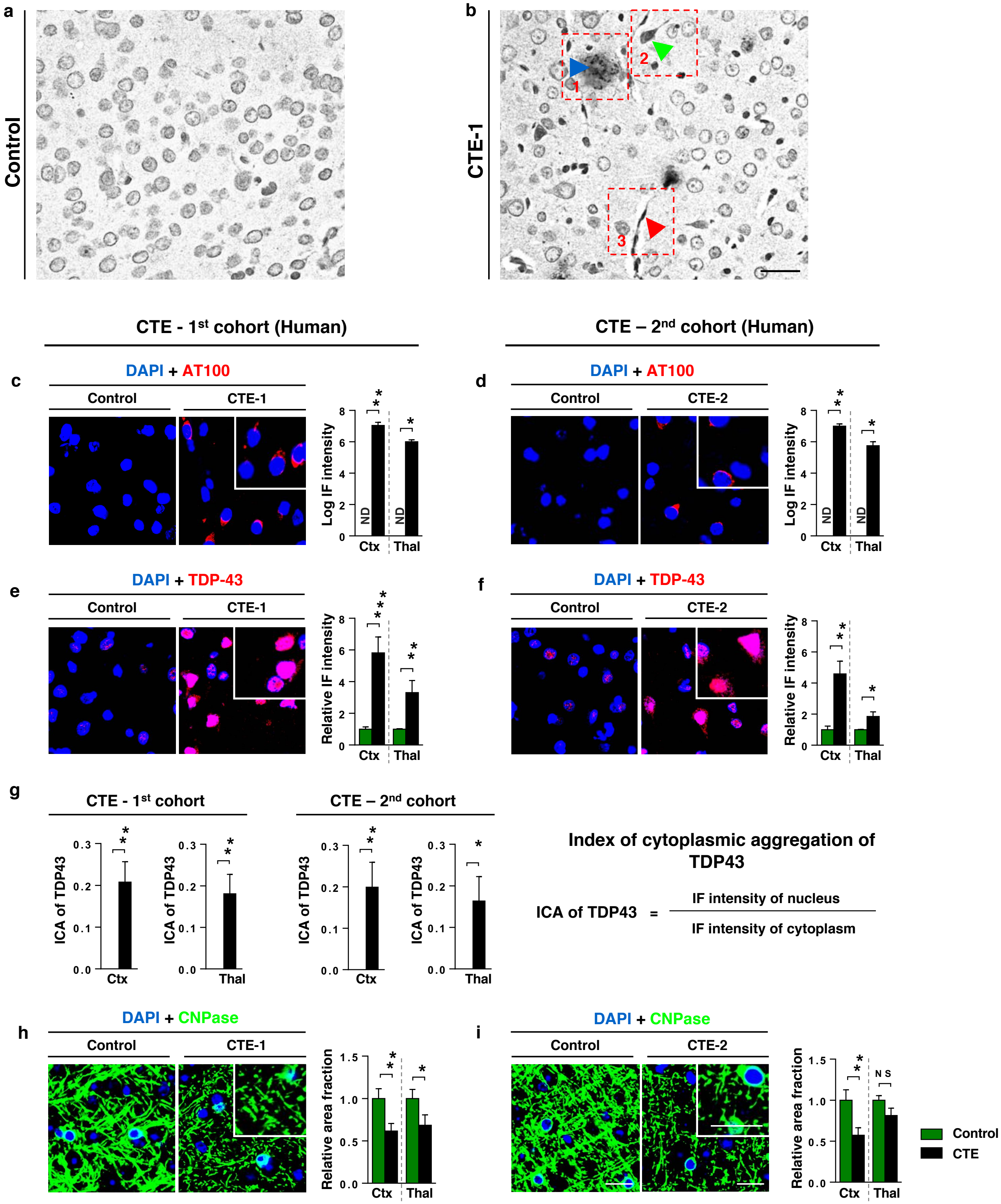

Supplementary Figure 3: *Cis* P-tau correlates with secondary pathologies including axonal pathology, late-stage neurofibrillary tangle formation, TDP-43 pathology and demyelination in the brain of collusion sport athletes with CTE. (a, b) Gallyas silver staining of corresponding cortex of a CTE brain (right) and an age-matched control (left). Different types of pathology in the CTE brain include 1) senile plaques outside neurons (blue arrow), 2) "neurofibrillary tangles" in neurons (green arrow) and 3) axonal bulb also referred to as a "retraction ball" and "neuropil threads" in neurons (red arrow). Scale bar, 50 µm. **(c-i)** Late-tangle formation (AT100) **(c, d)**, TDP-43 pathology with increased mislocalization and spreading from the nucleus to cytoplasm (TDP-43) **(e-g)**, and demyelination (CNPase) **(h, i)** were detected using immunostaining in two different brain regions, followed by confocal microscopy. Microscope images correspond to the cortex. The index of cytoplasmic aggregation (ICA) of TDP-43 method was used to analyze cytoplasmic TDP-43 level in a cell, with data represented as a column graph **(g)**. Ctx: parasagittal cortex, Thal: thalamus. ND: not detectable; NS: not significant; Scale bar, 40 µm. Results are shown as mean ± S.E.M. and p values calculated using unpaired two-tailed parametric Student's t-test. *p<0.05, **p<0.01, ***p<0.001, ****p<0.0001.

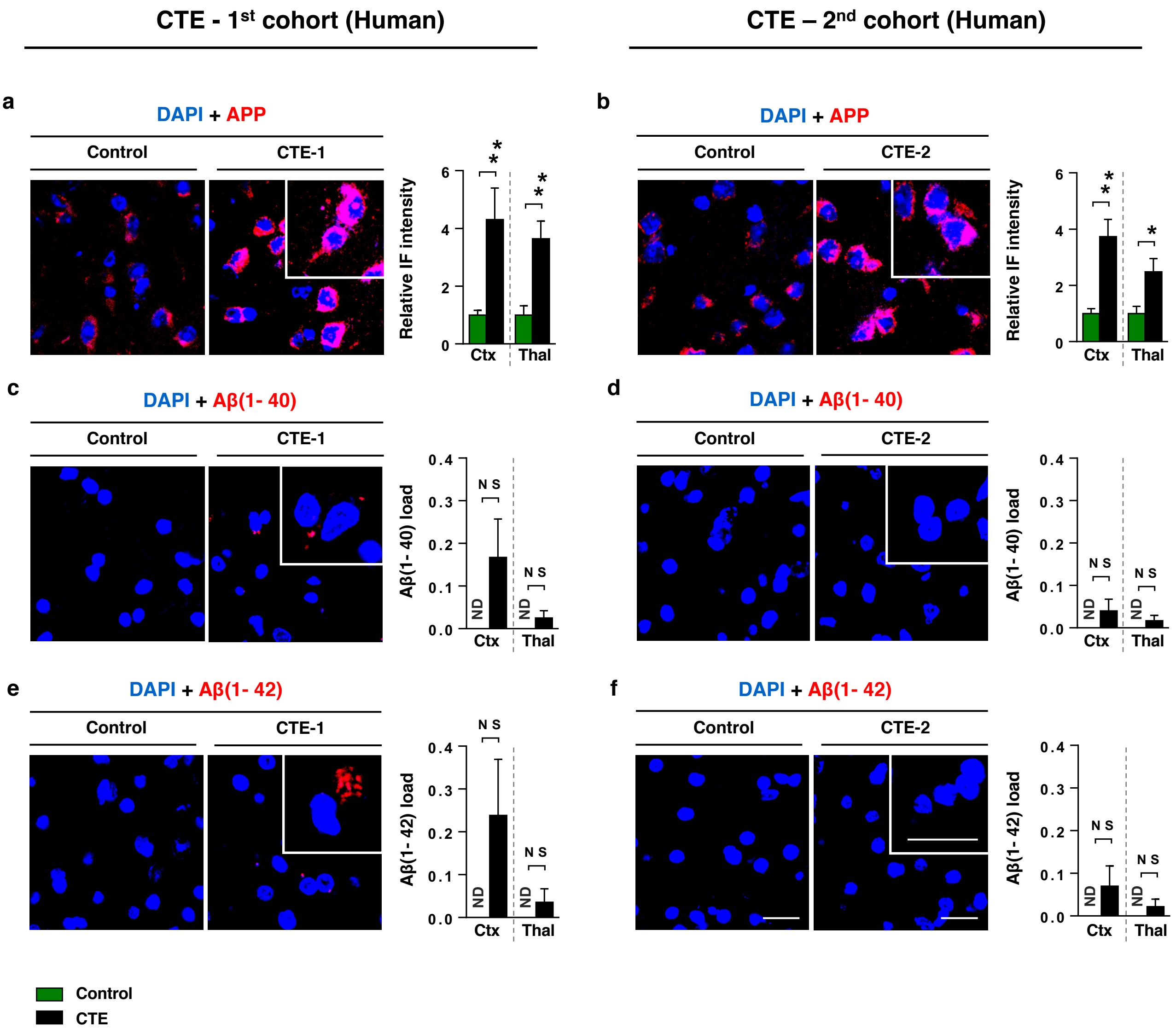

Supplementary Figure 4: CTE induced APP accumulation, without any significant beta-amyloid plaque formation. Increased APP accumulation **(a, b)**, without significant increase in amyloid-[beta] Aβ(1-40) **(c, d)** and Aβ(1-42) **(e, f)** peptide aggregates, was detected by immunostaining followed by confocal microscopy, in two different brain regions from separate cohorts of CTE brain tissues from contact sport athletes. ND, not detectable; NS, not significant; Scale bar, 40 µm. Ctx: parasagittal cortex, Thal: thalamus. N.D., not detectable. Results are shown as mean ± S.E.M. and p values calculated using unpaired two-tailed parametric Student’s t-test. *p<0.05, **p<0.01, ***p<0.001, ****p<0.0001.

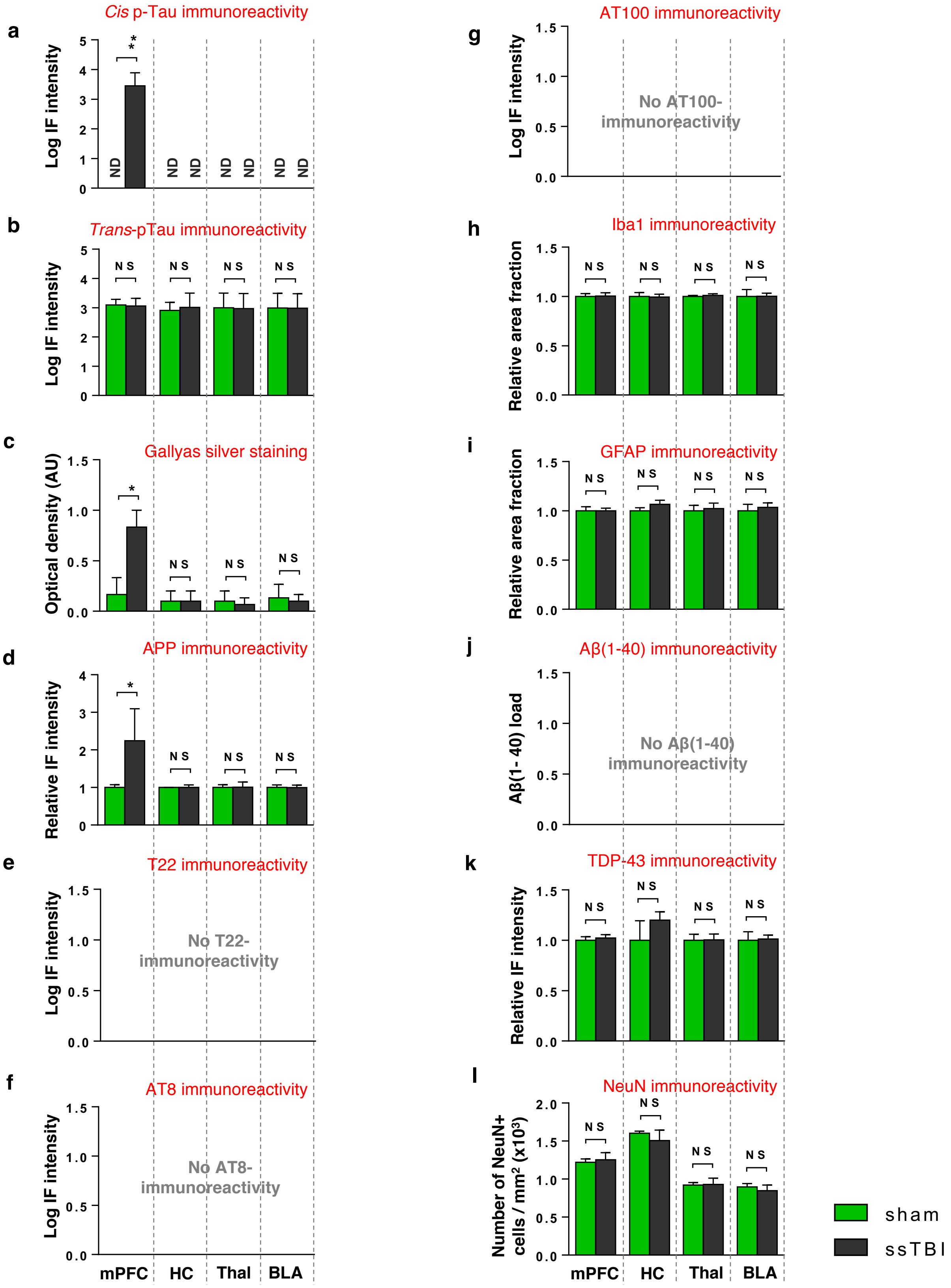

Supplementary Figure 5: *Cis* P-tau is an early pathological marker 48 hours after ssTBI. Increased *cis* P-tau **(a)**, but not *trans*, P-tau **(b)** was correlated with the presence of axonal pathology as detected by Gallyas silver staining **(c)**, together with increased APP accumulation **(d)** as detected by IF followed by confocal microscopy, in various brain regions of mice over 48 hours after ssTBI. There were not obvious changes in following secondary disease mechanisms, including oligomeric tau detected by T22 antibody **(e)**; early tangle detected by AT8 antibody **(f)**; late tangle detected by AT100 **(g)**; astrogliosis and microgliosis detected by GFAP and Iba1 antibody, respectively (h, i); beta-amyloid plaques detected by Aβ (1-40) **(j)**; TDP-43 pathology detected by TDP-43 antibody **(k)**; and NeuN+ neuronal loss **(l)** in various brain regions of mice over 48 hours after ssTBI. mPFC: medial prefrontal cortex, HC: hippocampus, Thal: thalamus, BLA: basolateral amygdala. ND, not detectable; NS, not significant. 4-5 WT male mice in immunohistochemistry studies per group; The data are presented as means ±SEM. The P-values were examined using unpaired two-tailed parametric Student's t-test. *p<0.05, **p<0.01, ***p<0.001, ****p<0.0001.

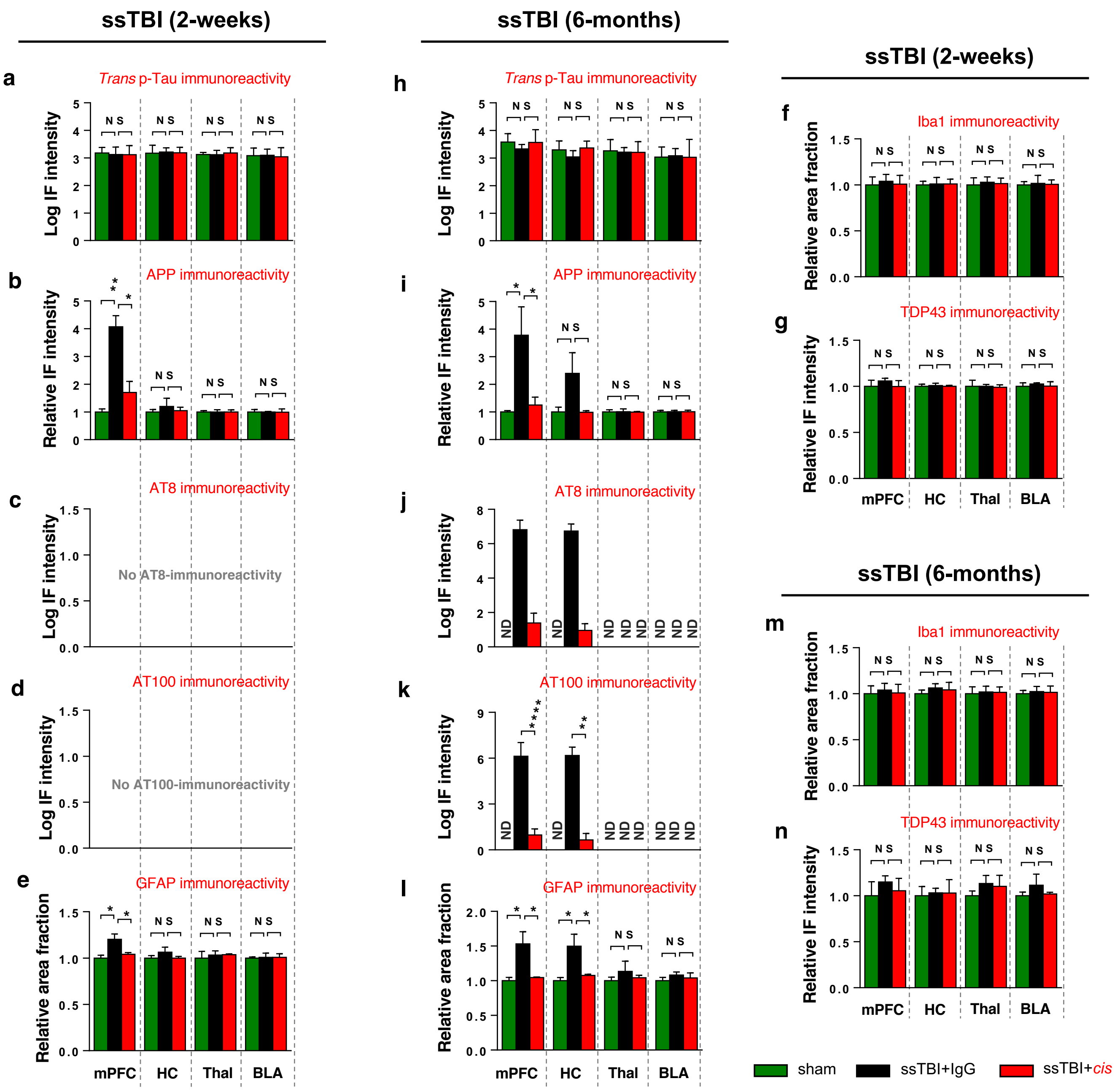

Supplementary Figure 6: Eliminating *cis* P-tau in ssTBI mice with *cis* mAb prevents the development and progression of a range of short- and long-term secondary pathologies after ssTBI. *Cis* mAb treatment eliminated APP accumulation **(b, i)** and astrogliosis **(e, l)** at 2 weeks **(b, e)** and their spreading to hippocampus at 6 months **(i, l)**. There were not early tangles (AT8) **(c)** or late tangles (AT100) **(d)** over two-weeks, but both early tangles (AT8) **(j)** or late tangles (AT100) **(k)** were observed at 6 months and eliminated by cis mAb treatment, as detected by immunostaining, followed by confocal microscopy. There are not differences in trans P-tau intensity **(a, h)**, Iba1+ area fraction **(f, m)** and TDP-43 intensity **(g, n)** at 2 weeks or 6 months after ssTBI. mPFC: medial prefrontal cortex, HC: hippocampus, Thal: thalamus, BLA: basolateral amygdala. ND, not detectable; NS, not significant. The brains of 4-5 WT male mice were examined in immunohistochemistry studies per group; The data were presented as means ±SEM. The P-values were examined using unpaired two-tailed parametric Student's t-test. *p<0.05, **p<0.01, ***p<0.001, ****p<0.0001.

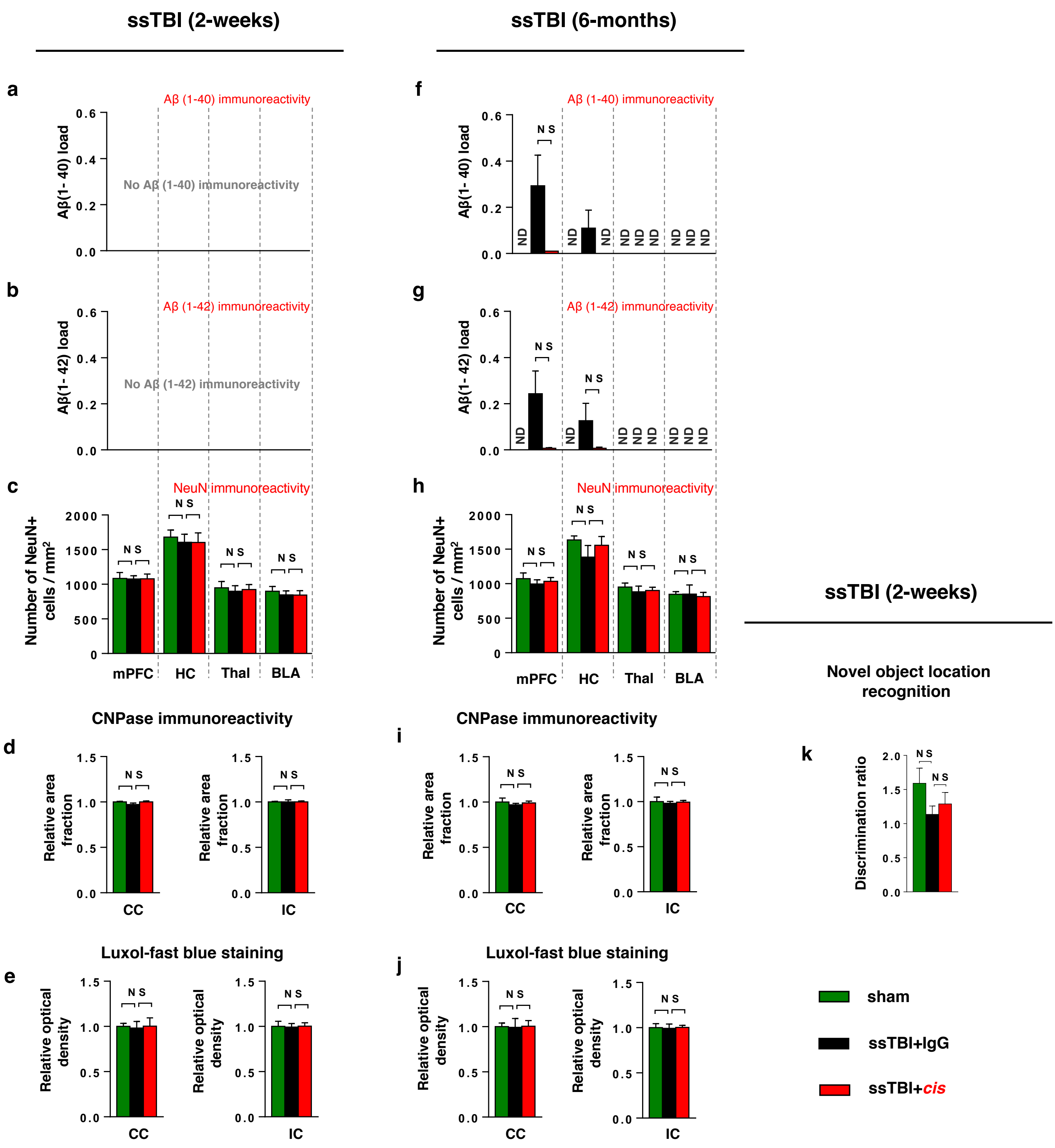

Supplementary Figure 7: There is no obvious beta-amyloid plaque formation or demyelination at 2 weeks or 6 months after ssTBI. There were not significant changes in amyloid-[beta] Aβ(1-40) **(a, f)**, Aβ(1-42) **(b, g)** aggregates, the number of NeuN-positive neurons **(c, h)**, or myelination as detected by CNPase **(d, i)** or Luxol-fast blue staining **(e, j)** between sham and the two TBI groups at 2 weeks or 6 months after ssTBI. **(k)** There was not significant difference in novel location recognition memory performance between sham and the two TBI groups at 2 weeks. mPFC: medial prefrontal cortex, HC: hippocampus, Thal: thalamus, BLA: basolateral amygdala, CC: corpus callosum, IC: internal capsule. ND, not detectable; NS, not significant. The brains of 4-5 WT male mice were examined in immunohistochemistry udies per group; The data are presented as means ±SEM.

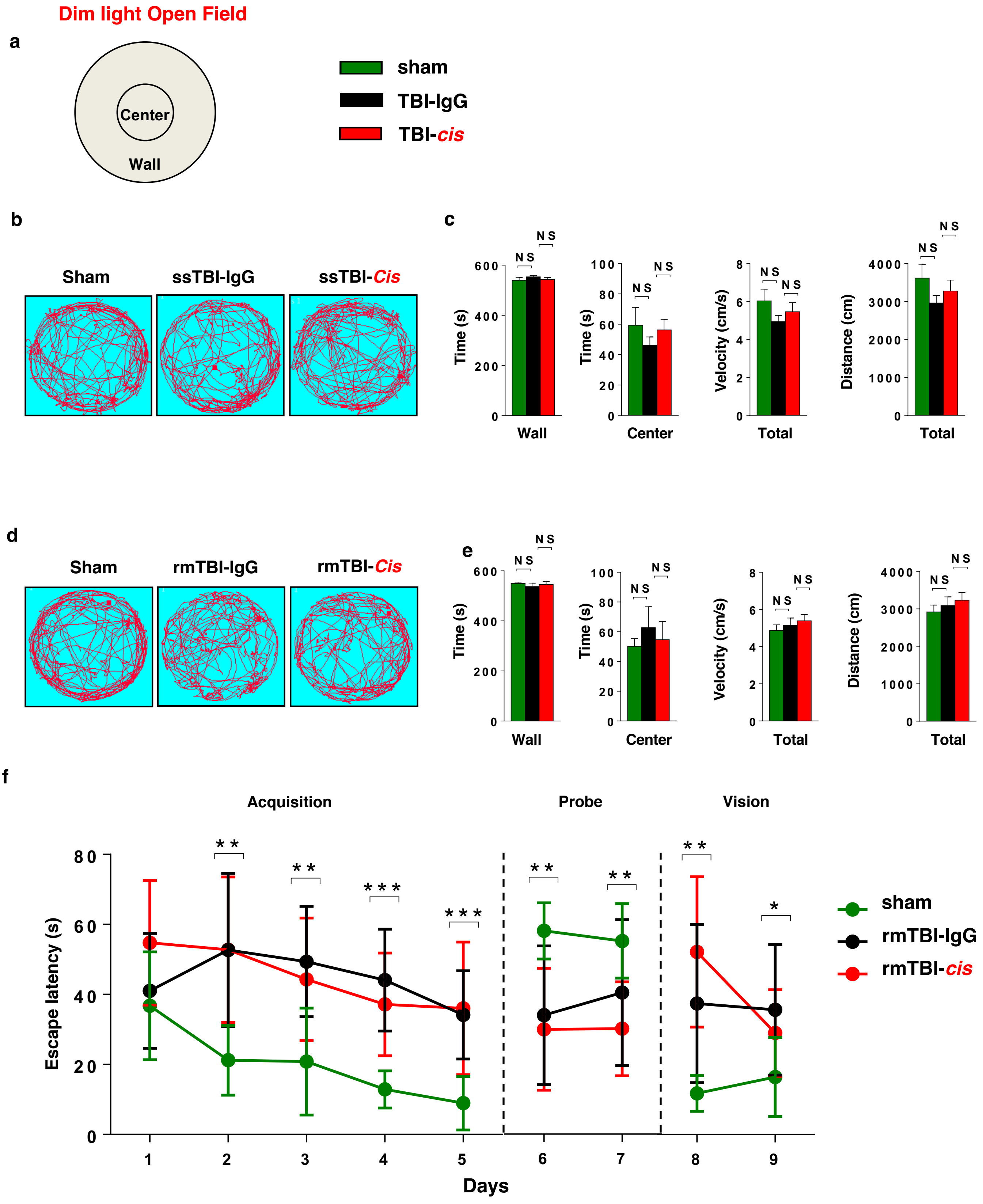

Supplementary Figure 8: There are no significant differences in baseline exploratory/locomotor activity 6 months after ssTBI and rmTBI. There were not significant differences in dim-light (30-50 lux) open field activity **(a)** 6 months after ssTBI **(b, c)** or rmTBI **(d, e)**. *Cis* mAb treatment did not improve the performance on the Morris water maze at 6 months after rmTBI **(f)**. NS, not significant. 9-10 WT male mice were subjected to behavioral studies per group. The data are presented as means ±SEM (a-e) or ±SD (f). The P-values were calculated using unpaired two-tailed parametric Student's t-test or two-way ANOVA followed by Bonferroni's t-test. *p<0.05, **p<0.01, ***p<0.001.

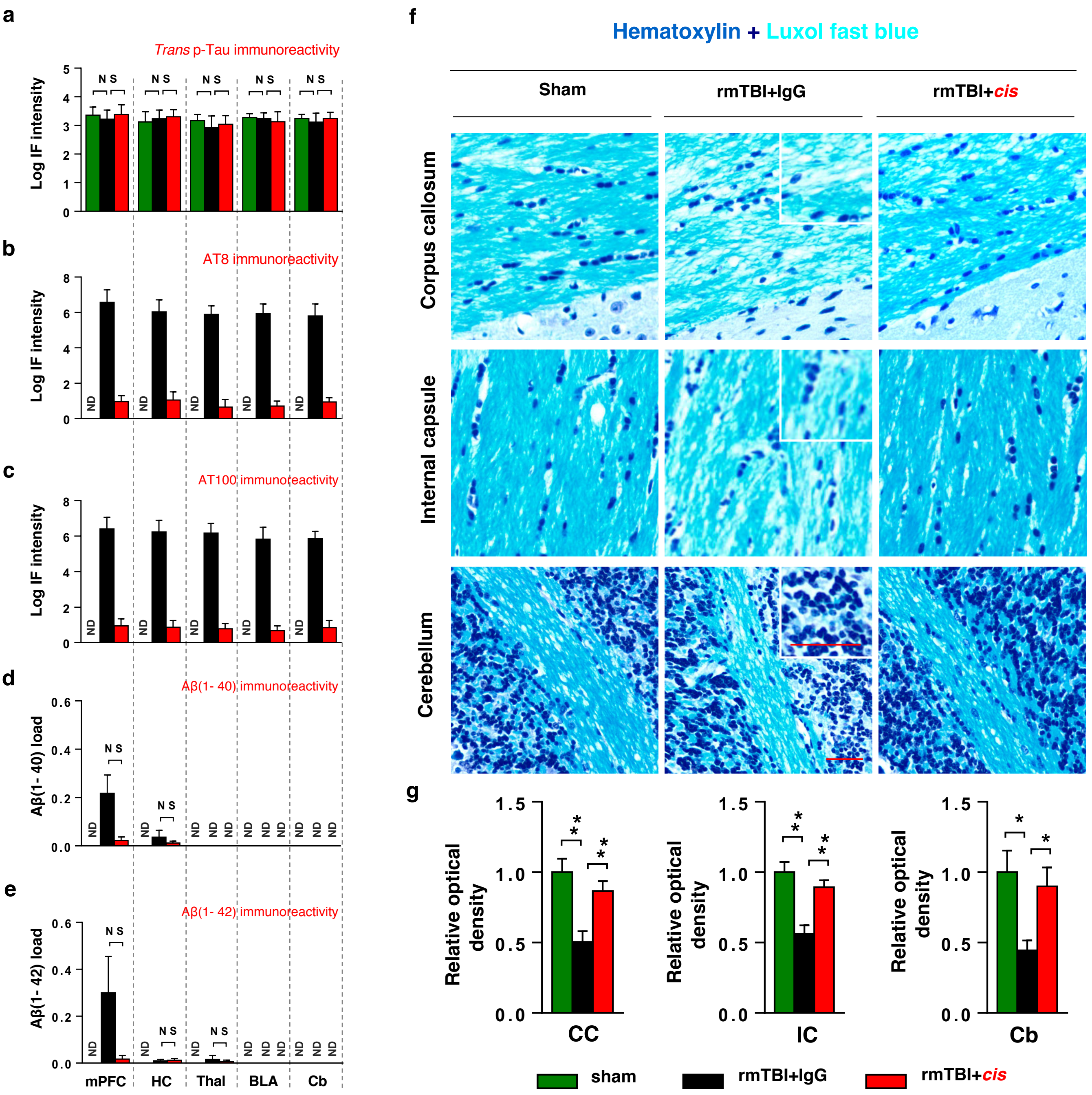

Supplementary Figure 9: Eliminating *cis* P-tau in rmTBI mice with *cis* mAb prevents the development and progression of a range of secondary pathologies 6 months after injury. Mice were subjected to 7 mild TBI events over 9 days and were treated with *cis* mAb or IgG isotype control over 4 months before being subjected to phenotypic analyses at 6 months. *Cis* mAb treatment prevented the development and spreading of early tangle formation AT8 **(b)** and late tangle formation AT100 **(c)** without changing *trans* P-tau **(a)** in various brain regions, as detected by IF followed by confocal microscopy. There were not significant changes in amyloid-[beta] Aβ(1-40) **(d)** and Aβ(1-42) **(e)** aggregates at 6 months after rmTBI. *Cis* mAb treatment prevented the development of demyelination in the different brain regions as detected by Luxol fast blue staining **(f, g)**. Inset microscope images are the high magnification image of selected area denoted by the white. Scale bar, 40 µm. mPFC: medial prefrontal cortex, HC: hippocampus, Thal: thalamus, BLA: basolateral amygdala, CC: corpus callosum, IC: internal capsule, and Cb: cerebellum. N.D., not detectable; NS, not significant. Brains of 4-5 WT male mice were used in immunohistochemistry studies per group; The data are presented as means ±SEM. The P-values were calculated using unpaired two-tailed parametric Student's t-test. *p<0.05, **p<0.01, ***p<0.001, ****p<0.0001.

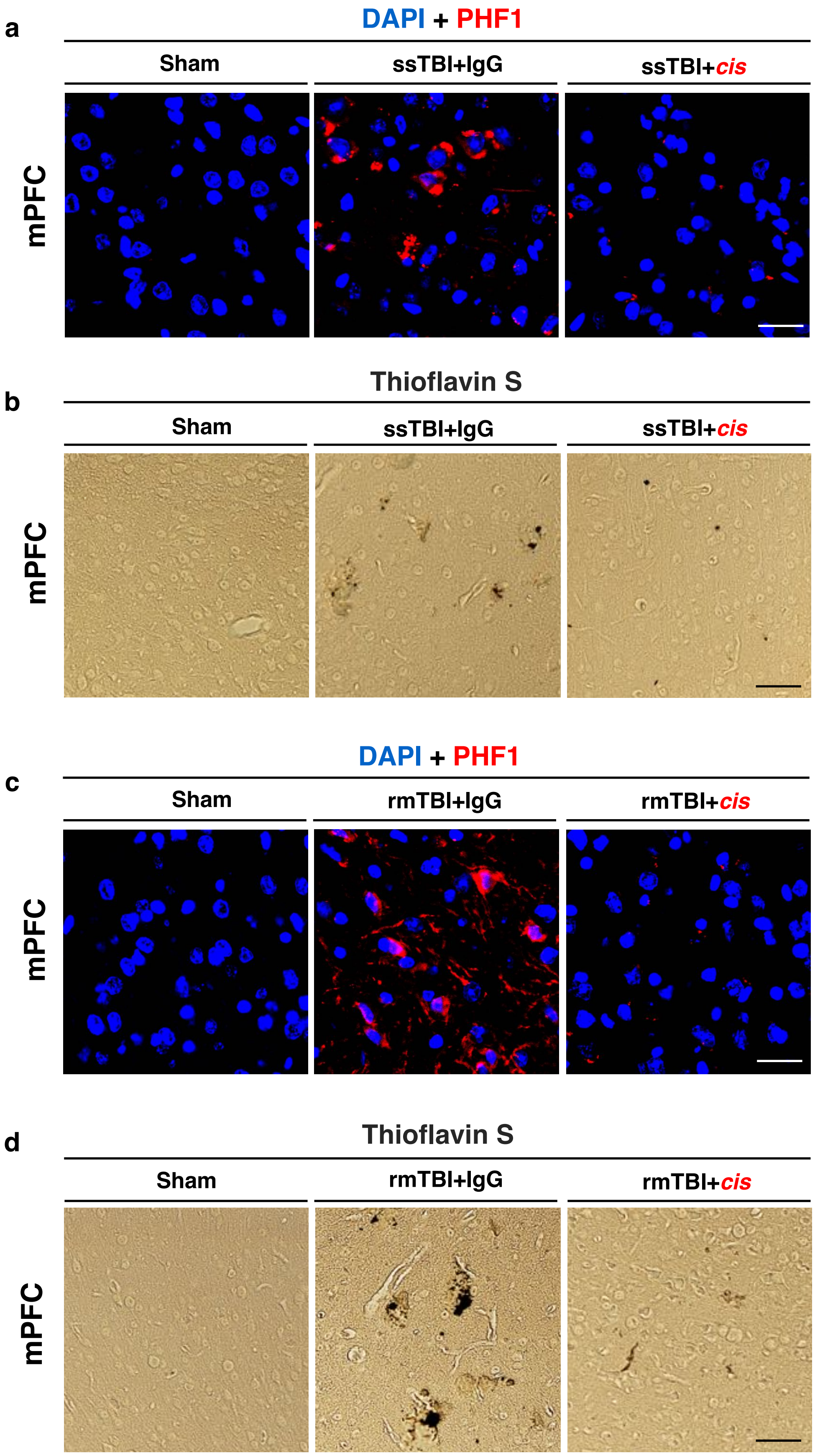

Supplementary Figure 10: Neutralization of *cis* P-tau by cis mAb effectively eliminates *cis* P-tau induction, but also prevents the development of tangle-like pathologies in chronic phases of ssTBI and rmTBI. Mice were subjected to ssTBI or rmTBI and were treated with *cis* mAb or IgG isotype control over 4 months before being subjected to pathological analyses at 6 months. *Cis* mAb treatment prevented the development of tangle-like pathologies detected by PHF-1 and ThioS in mPFC of **(a, b)** ssTBI and **(c, d)** rmTBI mice, as visualized by confocal microscopy. Scale bar, 40 µm. mPFC: medial prefrontal cortex.

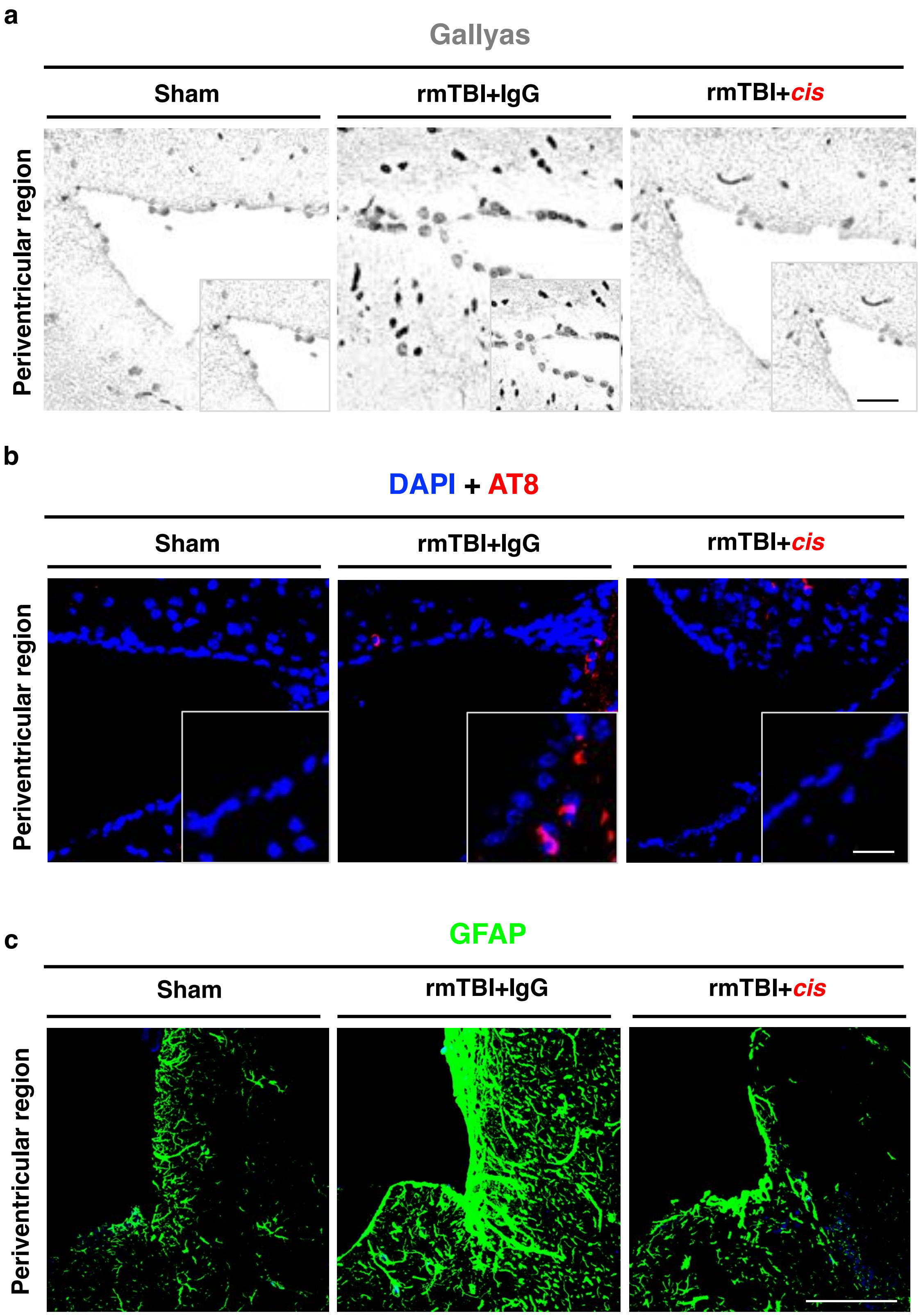

Supplementary Figure 11: Eliminating *cis* P-tau in rmTBI mice with *cis* mAb prevents the development of periventricular tangle-like formation and astroglial accumulation in chronic phases of rmTBI. Mice were subjected to rmTBI and were treated with *cis* mAb or IgG isotype control over 4 months before being subjected to pathological analyses at 6 months. *Cis* mAb treatment prevented the development of periventricular tangle-like formation detected by **(a)** Gallyas silver staining and **(b)** AT8 (early tangle) staining; and periventricular astrogliosis detected by **(c)** GFAP staining in mPFC of rmTBI mice, as visualized by confocal microscopy. Scale bars: 40 & 80 µm.

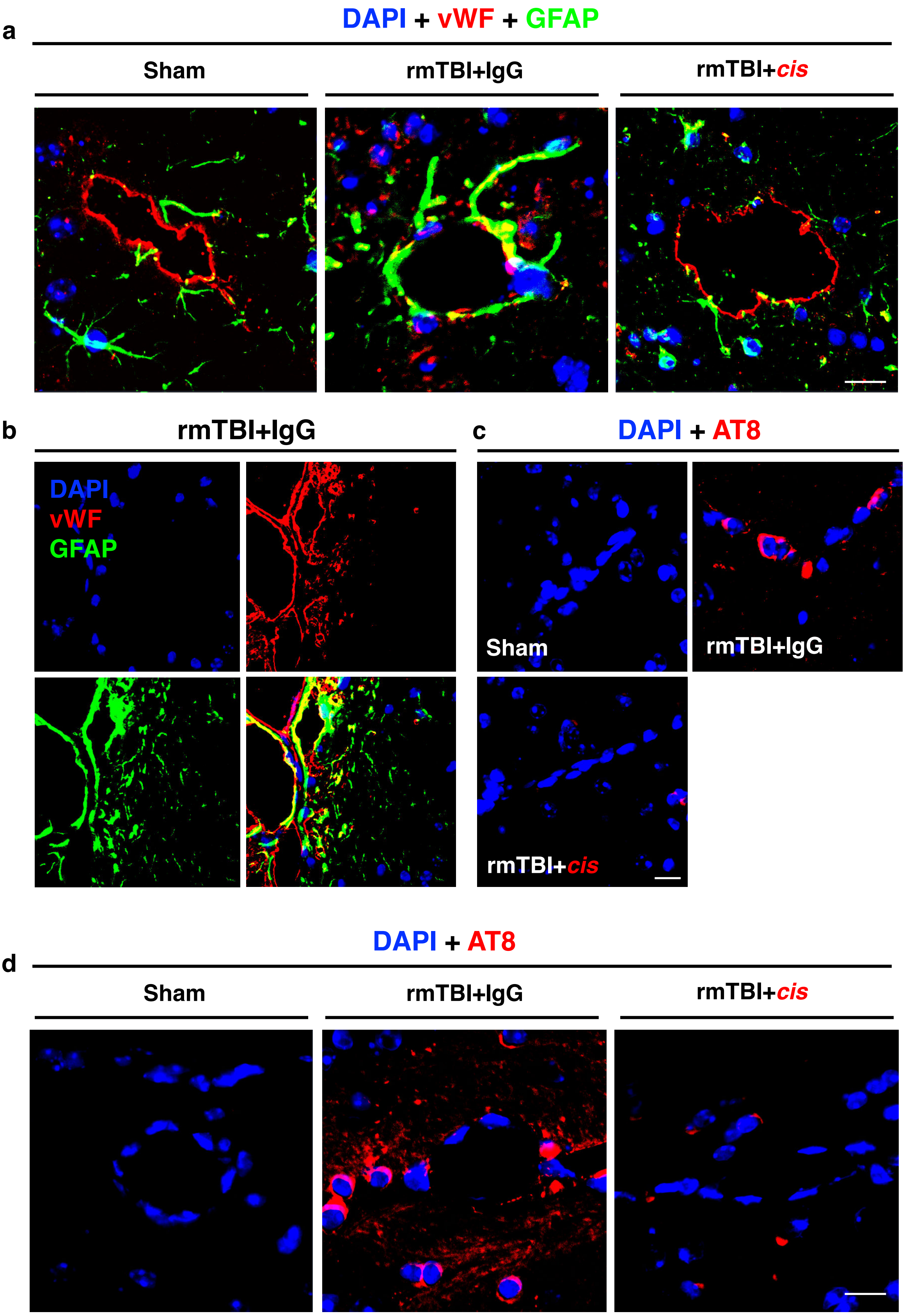

Supplementary Figure 12: Eliminating *cis* P-tau in rmTBI mice with *cis* mAb prevents the induction of (a, astrocytosis and neuronal-tangle accumulation at the perivascular elements and around small blood vessels in rmTBI animals. Mice were subjected to rmTBI and were treated with *cis* mAb or IgG isotype control over 4 months before being subjected to pathological analyses at 6 months. *Cis* mAb treatment prevented the development of **(a, b)** astrocytosis detected by GFAP (astrocyte) and Von Willerbrand factor (endothelial cells) and **(c, d)** tangle-like formation detected by AT8 (early tangle) at the perivascular elements and around small blood vessels in rmTBI animals, as visualized by confocal microscopy. Scale bars: 40 & 80 µm.

Figure 2a

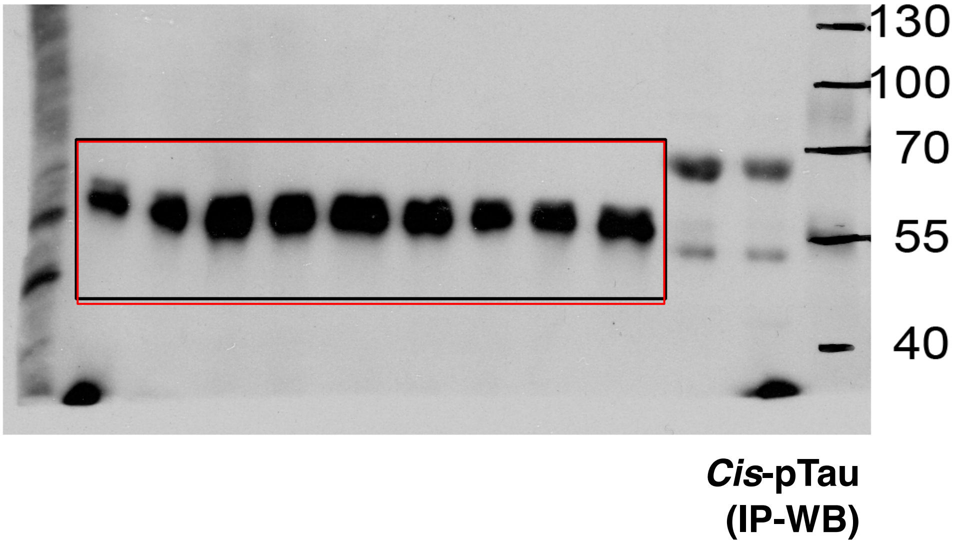

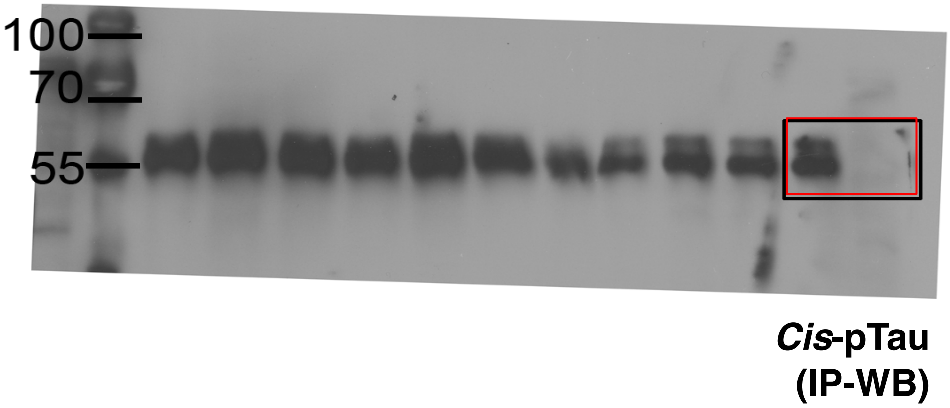

Supplementary Figure 13: Uncropped western blot images for figure 2a. Molecular weight (kDa) of the pre-stained protein ladder bands are indicated on the right and left side of the panels, respectively. Antibodies used to probe the nitrocellulose membranes are indicated on the bottom right. Rectangles represent the cropped images shown in figures.

Figure 4c

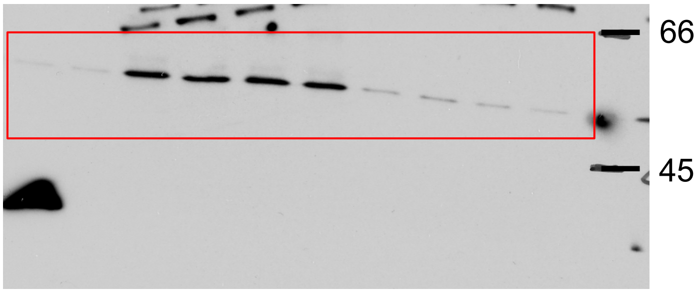

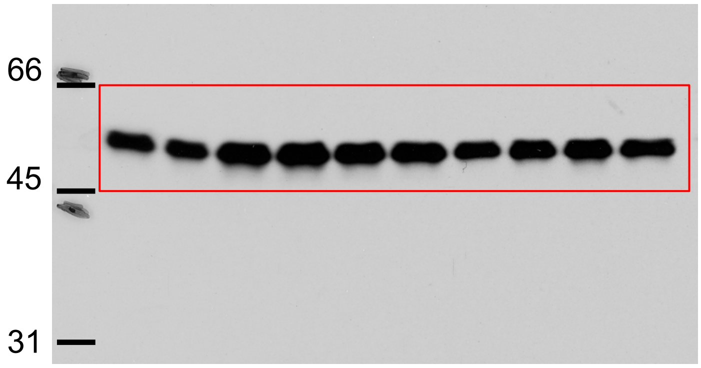

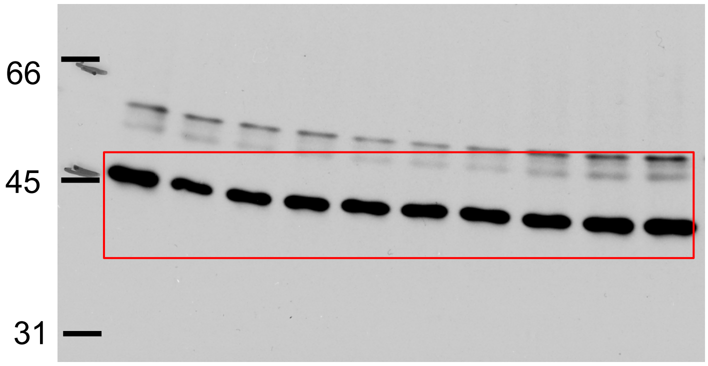

Supplementary Figure 14: Uncropped western blot images for figure 4c. Molecular weight (kDa) of the pre-stained protein ladder bands are indicated on the left or right side of the panels. Antibodies used to probe the nitrocellulose membranes are indicated on the bottom right. Rectangles represent the cropped images shown in figures.

Figure 5b

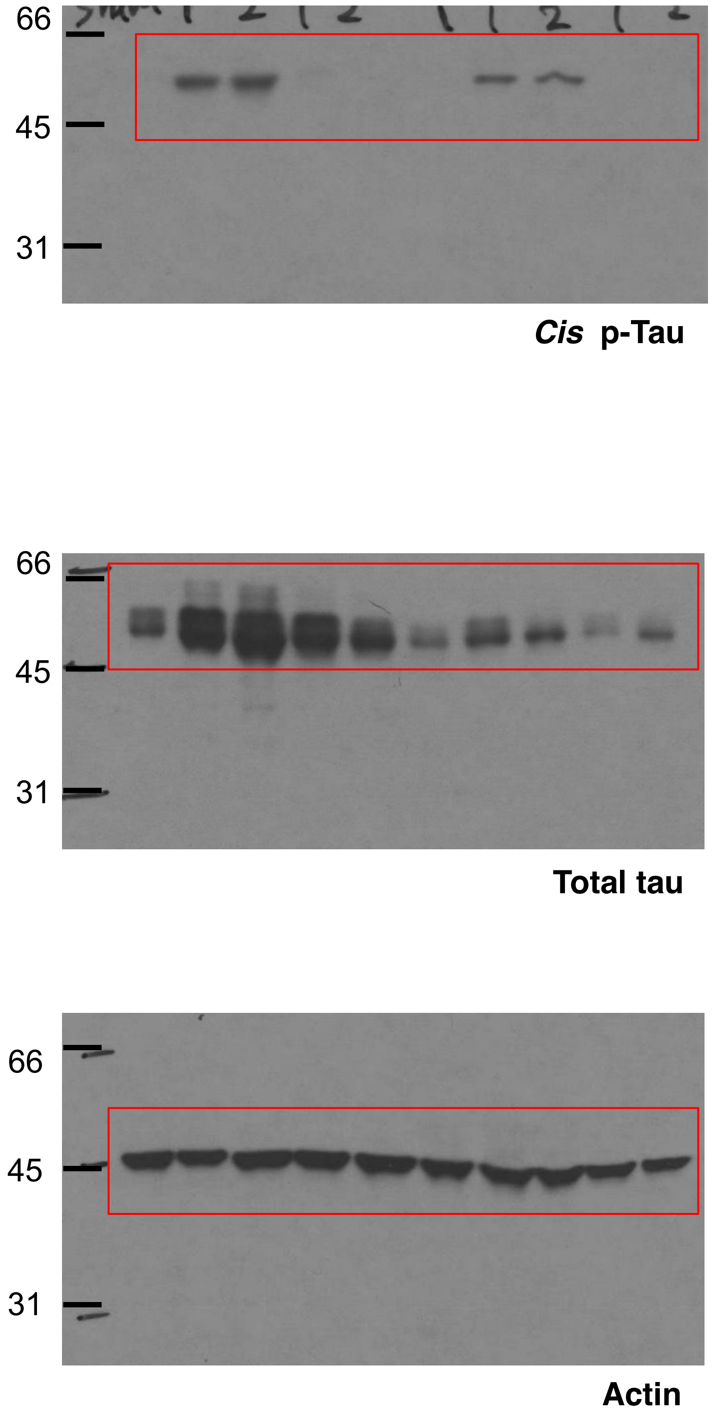

Supplementary Figure 15: Uncropped western blot images for figure 5b. Molecular weight (kDa) of the pre-stained protein ladder bands are indicated on the left or right side of the panels. Antibodies used to probe the nitrocellulose membranes are indicated on the bottom right. Rectangles represent the cropped images shown in figures.

Figure 6b

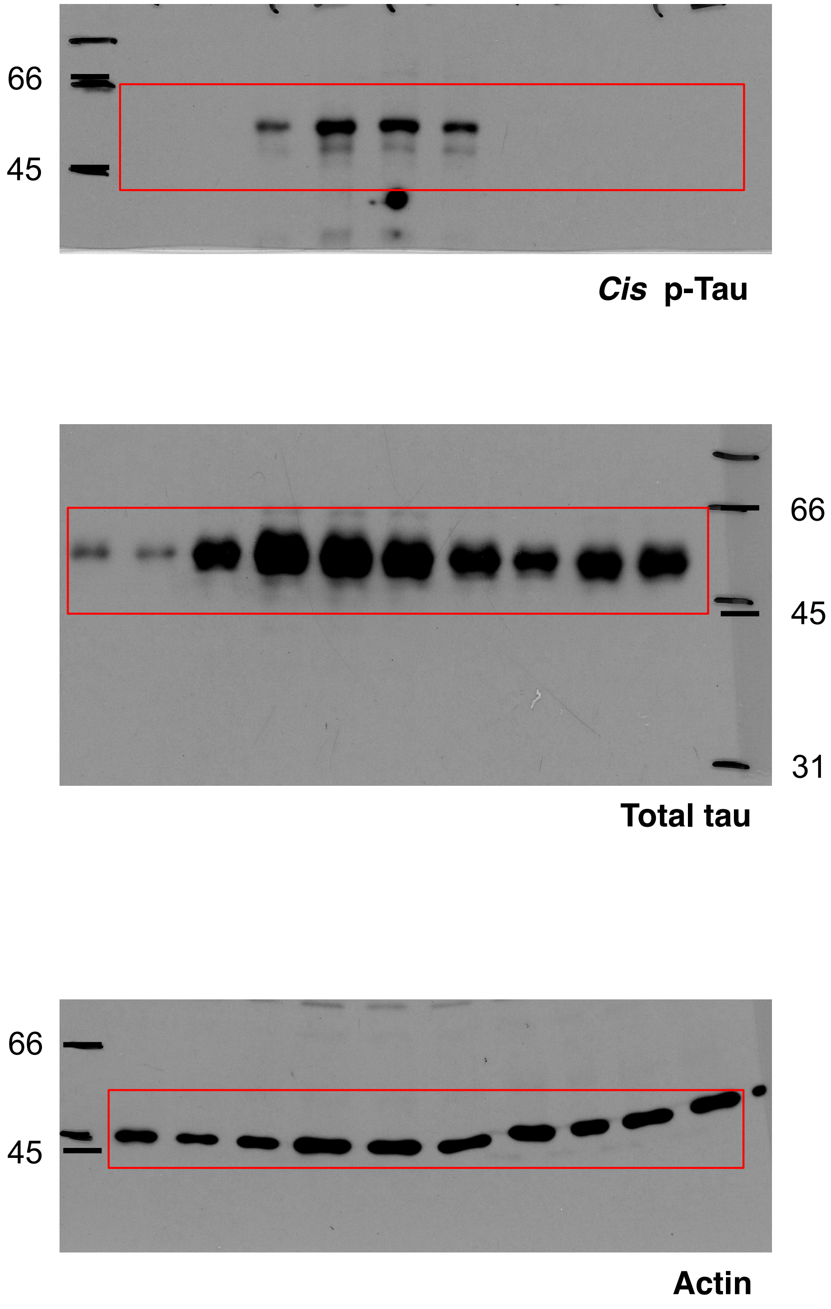

Supplementary Figure 16: Uncropped western blot images for figure 6b. Molecular weight (kDa) of the pre-stained protein ladder bands are indicated on the left or right side of the panels. Antibodies used to probe the nitrocellulose membranes are indicated on the bottom right. Rectangles represent the cropped images shown in figures.

Figure 7c

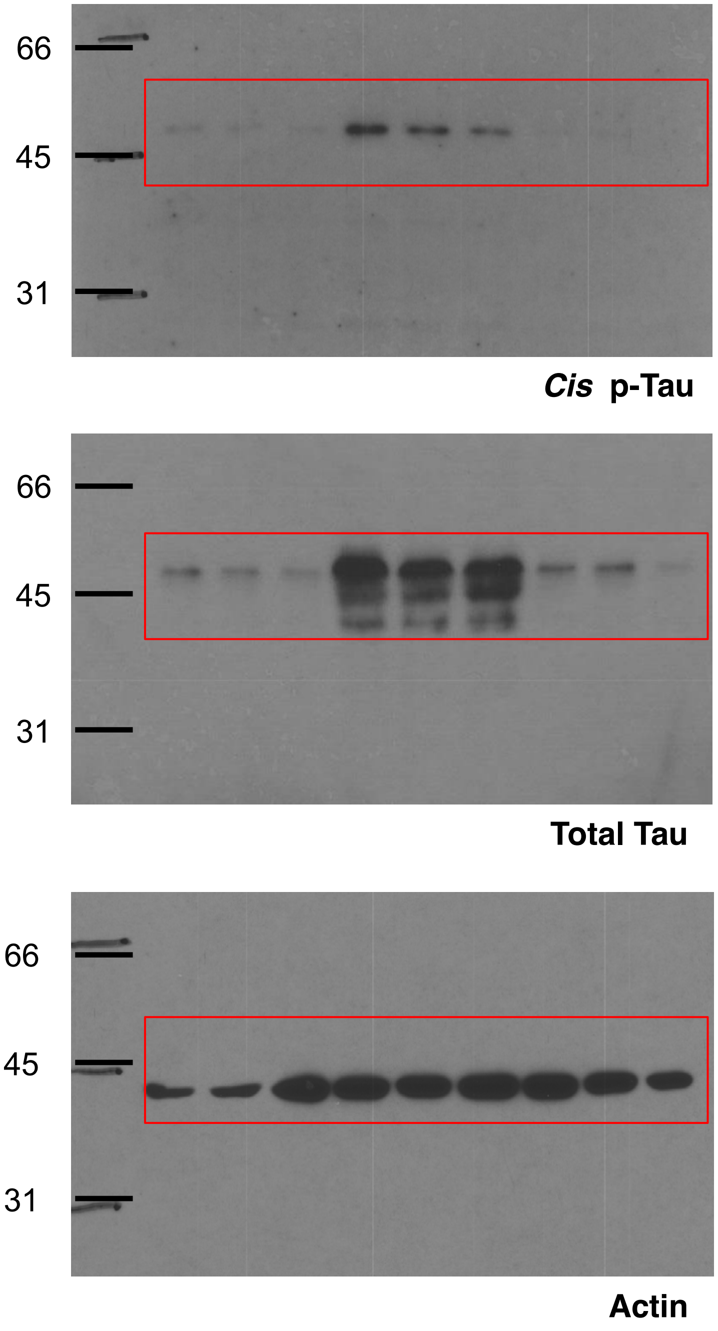

Supplementary Figure 17: Uncropped western blot images for figure 7c. Molecular weight (kDa) of the pre-stained protein ladder bands are indicated on the left side of the panels. Antibodies used to probe the nitrocellulose membranes are indicated on the bottom right. Rectangles represent the cropped images shown in figures.

Table S1. The clinical information and pathologies of fatal human TBI brains

Patient No	Age (Y)	TBI cause	Survival time	Brain region	*Cis* P-tau	T22	AT8	AT100	Aβ	TDP-43
1	51	Motor vehicle accident	1 hour	Cortex	0	0	0	0	0	0
				Hippocampus	+/-	+/-	+/-	0	0	0
2	19	Motor vehicle accident	8 hours	Cortex	+	0	0	0	0	0
				Hippocampus	0	0	0	0	0	0
3	3	Motor vehicle accident	18 hours	Cortex	+	0	0	0	0	0
				Hippocampus	0	0	0	0	0	0
4	9	Motor vehicle accident	1 day	Cortex	++	0	0	0	0	0
				Hippocampus	0	0	0	0	0	0
5	40	Motor vehicle accident	2 days	Cortex	++	0	0	0	+	0
				Hippocampus	0	0	0	0	+	0
6	9	Motor vehicle accident	1 week	Cortex	++	0	0	0	0	0
				Hippocampus	0	0	0	0	0	0
7	38	Assault	15 hours	Cortex	++	0	0	0	0	0
				Hippocampus	0	0	0	0	0	0
8	37	Assault	1 day	Cortex	++	0	0	0	0	0
				Hippocampus	0	0	0	0	0	0
9	30	Assault	14 days	Cortex	+	0	0	0	0	0
				Hippocampus	0	0	0	0	0	0
10	40	Assault	14 days	Cortex	+	0	0	0	0	0
				Hippocampus	0	0	0	0	0	0
11	67	Fall	5 days	Cortex	++	0	0	0	0	0
				Hippocampus	+/-	+/-	+/-	0	0	0
12	65	Fall	7 days	Cortex	+	0	0	0	0	0
				Hippocampus	0	0	0	0	0	0
13	19	Fall	1 month	Cortex	+	0	0	0	0	0
				Hippocampus	0	0	0	0	0	0
14	26	Unknown	1 day	Cortex	++	0	0	0	0	0
				Hippocampus	0	0	0	0	0	0

0: Non-detectable; +/-, barely detectable; +, readily detectable; ++, strongly detectable.

Table S2. CSF *cis* P-tau levels correlate with functional outcome of TBI patients at 1 year after injury.

Patient No	Age (Y)	Gender	Initial GCS	1 year GOS*	CSF *cis* P-tau† (Mean±SD)	Source§
1029	18	Male	6	3	0.812±0.111	UPITT
1052	20	Male	7	1	1.041±0.187	UPITT
1073	21	Male	3	**	0.893±0.119	UPITT
1077	24	Male	3	1	1.102±0.158	UPITT
1086	59	Male	7	1	1.025±0.170	UPITT
1092	50	Female	7	**	1.308±0.114	UPITT
1095	45	Male	6	1	0.884±0.173	UPITT
1097	26	Male	7	5	0.743±0.046	UPITT
1098	19	Male	7	5	0.797±0.169	UPITT
1099	35	Male	7	3	0.955±0.110	UPITT
1101	46	Male	6	5	0.655±0.121	UPITT
1103	20	Male	7	**	0.653±0.120	UPITT
1106	16	Male	7	5	0.914±0.017	UPITT
1115	55	Female	8	3	0.694±0.012	UPITT
1117	27	Male	7	3	0.847±0.011	UPITT
1118	27	Male	8	1	0.810±0.007	UPITT
1121	26	Male	8	**	0.639±0.020	UPITT
1123	17	Male	6	3	0.980±0.037	UPITT
1124	57	Male	7	**	0.720±0.018	UPITT
1125	32	Male	7	1	0.840±0.002	UPITT
1	29	Male	6	5	0.726±0.059	HMS
15	30	Female	5	**	0.925±0.047	HMS
18	72	Female	7	1	0.637±0.026	HMS
19	23	Male	7	5	0.550±0.120	HMS
20	34	Male	3	5	0.696±0.115	HMS
22	24	Female	3	5	0.876±0.043	HMS

* Glasgow Outcome Scale (GOS) values were assessed at 1 year after TBI
** GOS values are not available because those patients are lost to follow up.
†*Cis* P-tau levels in the CSF samples of TBI patients at 4-6 days after injury were assayed in triplicates using direct ELISA and presented in values of absorption (OD405 nm).
§Samples were obtained from University of Pittsburgh (UPITT), Pittsburgh, PA, or Brigham and Women Hospital, Harvard Medical School (HMS), Boston, MA.

Table S3. The clinical information of patients with CTE

1st CTE cohort

Patient No	Profession	Age (Y)	Primary diagnosis	Secondary diagnosis	Source
Control-1	-	59	No brain disease	-	UC *
Control-2	-	45	No brain disease	-	UC *
Control-3	-	26	No brain disease	-	UC *
CTE-1	NFL player	41	CTE	-	UC *
CTE-2	NFL player	36	CTE	-	UC *
CTE-3	Professional wrestler	40	CTE	-	UC *
CTE-4	NCAA Football player	74	CTE	AD	UC *

2nd CTE cohort

Patient No	Profession	Age (Y)	Primary diagnosis	Secondary diagnosis	Source
Control-1	-	61	No brain disease	-	BU**
Control-2	-	68	No brain disease	-	BU**
Control-3	-	67	No brain disease	-	BU**
CTE-1	NFL player	67	CTE	-	BU**
CTE-2	NFL player	50	CTE	-	BU**
CTE-3	Professional boxer	40	CTE	-	BU**
CTE-4	Professional boxer	50	CTE	-	BU**

*Department of Neurosurgery, University of Chicago Pritzker School of Medicine, Evanston, IL
**The CTE Center, Boston University School of Medicine, Boston, MA, These samples were used in Kondo et al., 2015, Nature, 523: 431-436.

Table S4. Combined analysis of the therapeutic effects of *cis* mAb treatments on the histopathological and functional outcomes in ssTBI mice and rmTBI mice

**_Cis_ mAb treatments of TBI mice**	
2 TBI mechanisms	Single severe TBI<br>Repetitive mild TBI
6 treatment regimens used	Short-term 10 day treatment<br>Long-term 4 month treatment<br>IP injections only<br>IP+ICV injections<br>Immediate treatment<br>4 hr delayed treatment<br>8 hr delayed treatment
Factor loading analysis method	Use conventional factor loading cut-off of 0.3 to determine variable retention for behavior construct factor(s);<br>Employ linear regression with the latent behavior construct factor(s) as the outcome, treatment as the predictor
**Therapeutic effects on histopathological outcomes**	
39 animals included in final factor analysis	13 for Sham group<br>13 for TBI group treated with IgG<br>13 for TBI group treated with *cis* mAb
14 histopathological outcomes (7 histopathological outcomes at two brain regions: cortex and hippocampus) included in final factor construct (correlation > 0.3)	*Cis* P-tau<br>Gallyas silver staining (axonal injury)<br>T22 (tau oligomers)<br>AT8 (Early tangles)<br>AT100 (later tangles)<br>APP<br>GFAP
Difference between groups (*p* values)	Sham vs TBI-IgG: *p*=1.076e-10<br>TBI-IgG vs TBI-*cis* mAb: *p*=7.524e-07<br>Sham vs TBI-*cis* mAb: *p*=0.399
**Therapeutic effects on functional outcomes**	
99 animals included in final factor analysis	24 for Sham group<br>40 for TBI group treated with IgG<br>35 for TBI group treated with *cis* mAb
3 functional outcomes included in final factor construct (correlation > 0.3)	Ledge test<br>String-suspension<br>Voiding pattern
Difference between groups (*p* values)	Sham vs TBI-IgG: *p*=4.284e-06<br>TBI-IgG vs TBI-*cis* mAb: *p*=1.094e-5<br>Sham vs TBI-*cis* mAb: *p*=0.989

**Therapeutic effects on both histopathological and functional outcomes**	
36 animals included in final factor analysis	11 for Sham group<br>13 for TBI group treated with IgG<br>12 for TBI group treated with *cis* mAb
14 histopathological outcomes and<br>3 functional outcomes included in final factor construct (correlation > 0.3)	*Cis* P-tau<br>Gallyas silver staining (axonal injury)<br>T22 (tau oligomers)<br>AT8 (Early tangles)<br>AT100 (later tangles)<br>APP<br>GFAP<br>Ledge test<br>String-suspension<br>Voiding pattern
Difference between groups (*p* values)	Sham vs TBI-IgG: *p*=3.451e-05<br>TBI-IgG vs TBI-*cis* mAb: *p*=2.347e-05<br>Sham vs TBI-*cis* mAb: *p*=0.801
